# A High‐Density Raman Photometry for Tracking and Quantifying of AchE Activity in The Brain of Freely Moving Animals with Network

**DOI:** 10.1002/advs.202301004

**Published:** 2023-08-27

**Authors:** Zhonghui Zhang, Zhichao Liu, Peicong Wu, Xinhua Guo, Xiao Luo, Youjun Yang, Jinquan Chen, Yang Tian

**Affiliations:** ^1^ Shanghai Key Laboratory of Green Chemistry and Chemical Processes School of Chemistry and Molecular Engineering East China Normal University Dongchuan Road 500 Shanghai 200241 P.R. China; ^2^ State Key Laboratory of Precision Spectroscopy East China Normal University Dongchuan Road 500 Shanghai 200241 P.R. China; ^3^ State Key Laboratory of Supramolecular Structure and Materials College of Chemistry and Key Laboratory for Molecular Enzymology and Engineering of the Ministry of Education College of Life Science Jilin University Qianjin Road 2699 Changchun 130012 P.R. China; ^4^ State Key Laboratory of Bioreactor Engineering Shanghai Key Laboratory of Chemical Biology School of Pharmacy East China University of Science and Technology Meilong Road 130 Shanghai 200237 P.R. China

**Keywords:** acetylcholinesterase, biosensors, brain networks, fiber Raman

## Abstract

A high‐density Raman photometry based on a dual‐recognition strategy is created for accurately quantifying acetylcholinesterase (AchE) activity in 24 brain regions of free‐moving animals with network. A series of 5‐ethynyl‐1,2,3,3‐tetramethyl‐based molecules with different conjugated structures and substitute groups are designed and synthesized for specific recognition of AchE by Raman spectroscopy. After systematically evaluating the recognition ability toward AchE, 2‐(4‐((4‐(dimethylamino)benzoyl)oxy)styryl)‐5‐ethynyl‐1,3,3‐trimethyl‐3H‐indol‐1‐ium (ET‐5) is finally optimized for AchE determination, which shows the highest selectivity, the greatest sensitivity, and the fastest response time among the investigated seven molecules. More interestingly, using the developed probe for AchE with high accuracy and sensitivity, the optimized AchE regulated by nitric oxide (NO) is discovered for promoting the neurogenesis of neural stem cells (NSCs). Benefiting from the high‐density photometry, it is found that the activity and distribution of AchE varied in 24 brain regions, and the levels of AchE activity in 24 brain regions of Alzheimer's mice (AD) are lower than those of normal mice. It is the first time that a functional network of AchE in 24 brain regions is established. It is also found that the loss of AchE functional network in AD mice is restored and reconstructed by the controlled release of AchE regulated by NO.

## Graphical abstract

A high‐density Raman fiber photometry with a highly selective and sensitive probe is created for accurately quantifying and controllable regulating of AchE activity in NSCs and mouse brains. Using powerful tools, it is discovered that NO‐promoted neurogenesis of NSCs is regulated by AchE activity, which further treats AD through reconstructing the correlations of AchE activity between adjacent brain regions.

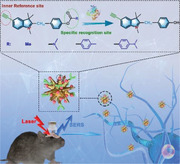

## 1 Introduction

Acetylcholinesterase (AChE) is an extrinsic membrane‐bound enzyme that can be projected into synapses.^[^ ^1^ ^]^ The “classical” role of AchE in the termination of acetylcholine‐mediated neurotransmission is of great significance for the treatment of neurodegenerative diseases such as AD.^[^ ^2^ ^]^ However, the complexity of AchE gene regulation, different localization in the non‐cholinergic, and the diversity of its molecular forms strongly suggest that AchE has additional “non‐classical” functions.^[^ ^3^ ^]^ It has been reported that AChE can induce synapse formation and cell migration through a mechanism independent of its catalytic activity.^[^ ^4^ ^]^ Recently, the therapeutic effect of acetylcholinesterase inhibitors for AD based on cholinergic damage hypothesis has been widely questioned. Although it can improve cognitive impairment in AD patients, the treated patients have significant side effects and only short‐term efficacy.^[^ ^5^ ^]^ Thus, it is the key challenge to quantitative determination of AchE in the live brain for understanding the mechanism of effective treatment of AD diseases, thus identifying “classical” or “non‐classical” functions of AchE in the brain. Several elegant methods have been developed for quantitative detection of AchE activity, such as colorimetric method and fluorescent imaging.^[^ ^6^ ^]^ However, monitoring and quantification of AchE activity in the living brain of freely moving animals remain a challenge due to the complexity of the brain and the lack of appropriate in situ detection tools. Surface‐enhanced Raman spectroscopy (SERS) provides an optimal strategy for imaging and biosensing of chemical signals due to high spectral resolution and resistance to autofluorescence of molecular fingerprint information.^[^ ^7^ ^]^ We recently constructed a SERS opto‐physiological microarray probe for determination of extracellular signals in the cortex of a mouse brain.^[^ ^8^ ^]^ However, the relatively limited penetration of excitation light prevents current Raman probe from being applied to deep brain. More recently, we developed a Raman fiber photometry for simultaneously monitoring the levels of pH, calcium ion (Ca^2+^), and superoxide anion (O_2_^•−^)in the brain of a freely moving mouse.^[^ ^9^ ^]^

Herein, a high‐density Raman platform was established for quantifying AchE activity with high selectivity and accuracy, in 24 brain regions of freely moving mice. Firstly, a series of 5‐ethynyl‐1,2,3,3‐tetramethyl‐based molecules (ET‐R, *R =* 1–7) with different conjugated structures and substitute groups were designed and synthesized. By comparing the response‐ability and selectivity of these molecules, ET‐5 was finally selected as an optimized probe to determine AchE in vivo because it showed the strongest recognition ability, the highest sensitivity, and the fastest response time. Based on a dual‐recognition strategy through specific chemical reactions and characteristic fingerprint peaks of Raman spectroscopy, the developed biosensor recognized AchE with high selectivity. Furthermore, the stable SERS band of ET‐5 at 2031 cm^−1^ was selected as a reference element for built‐in correction, avoiding environmental effects, probe concentrations, and light sources. Using the optimized biosensor, we discovered that increasing activity of AchE regulated by gradual release of NO was favorable for promoting the proliferation and differentiation of NSCs. Meanwhile, high‐density Raman fiber photometry was built up with significantly improved efficiency of Raman signals collection by optimizing optical fiber parameters. The developed Raman fiber platform was first used for AchE activity detection in 24 brain regions of free‐moving mice. Using this powerful tool, it was found that the activity and distribution of AchE varied in 24 brain regions, and AchE activities significantly decreased in AD mice compared with those in normal brains. More importantly, using high‐density fiber Raman photometry, an AchE‐related functional network in 24 brain regions was established. AD mice showed a significant loss of functional networks (∼33%) compared with normal mice. We also found that the loss of AchE functional network in early AD mice can be reconstructed by the controlled release of AchE regulated by NO.

## 2 Results and Discussion

### 2.1 Design of SERS probe for specific recognition of AchE

**Figure 1:**
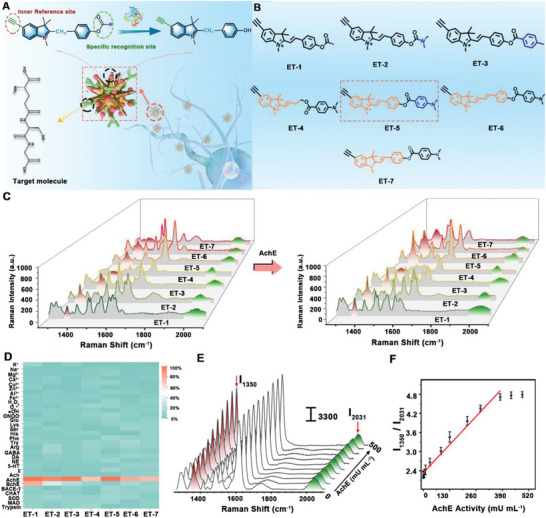
A) The designed efficiency AchE Raman detection platform for selective determination of the AchE activity. B) The corresponding molecular structures of ET‐R. C) Raman spectra of the investigated 7 molecules before and after addition of 500 mU mL^‐1^ AchE. D) Summarized data of selectivity for ET‐R (*R* = 1–7) toward various enzymes, neurotransmitters, amino acids, metal ions, and common ROS, respectively. The concentration of metal ions (except K^+^, Na^+,^ and Mg^2+^): 100 µM. The concentrations of K^+^, Na^+,^ and Mg^2+^ were 100 mM, 100 mM, and 300 µM, respectively. The concentration of ROS, amino acids, and neurotransmitters: 300 µM. The activity of enzymes: 200 mU mL^‐1^. E) Raman spectra of AuNSs@GSH@ET‐5 with the addition of AchE at various activities (0, 0.1, 0.5, 1, 2, 5, 10, 20, 50, 100, 200, 300, 400, 500 mU mL^‐1^). F) Calibration curve between Raman intensity ratio (I_1350_/I_2031_) and various activities of AchE in E) (*n* =10, S. E. M.).

As a starting point, gold nanostars (AuNSs) were synthesized as an effective SERS substrate. Transmission electron microscope (TEM) results demonstrated that the prepared nanostars with sharp tips were mono‐dispersed with size of 75 ± 5 nm (*n* = 50, S. D., Figure S1A,B, Supporting Information). Meanwhile, surface plasmon resonance (SPR) absorption peak of AuNSs was 780 nm (Figure S1C, Supporting Information). On the other hand, to specifically recognize AchE in the brain, a series of ET‐R with different conjugated structures and substitute groups were designed composed of four parts: 1,3,3‐trimethyl‐2‐styryl‐3H‐indol‐1‐ium structure improved polarization rate and accelerated binding to AchE through electrostatic interaction; substitute group (R) was synthesized for matching and combining the cavity of AchE; ester group for the enzymatic reaction with AchE; and free alkenyl for self‐assembling of ET‐R molecule onto the Raman substrate (**Figure** **1**A,B). All products were characterized by nuclear magnetic resonance (NMR), and high‐resolution mass spectroscopy (HR‐MS) (Figures S2–S38, Supporting Information). Furthermore, ET‐R and synapse‐targeted glutathione (GSH) were immobilized onto AuNSs surface, named AuNSs@GSH@ET‐R. A redshift (8–23 nm) of AuNSs@GSH@ET‐R (*R =* 1–7) was observed after GSH and ET‐R were assembled onto the AuNSs (Figure S39A, Supporting Information). In addition, dynamic light scattering (DLS) demonstrated that the diameter of AuNSs@GSH@ET‐R (*R =* 1–7) increased by 54±15 nm, compared with individual AuNSs (70±21 nm) (Figure S39B, Supporting Information). Moreover, Zeta potential of individual AuNSs was measured as 40.81±4.25 mV, while that of AuNSs@GSH@ET‐R (*R =* 1–6) changed to 21.8–26.6 mV. Interestingly, the Zeta potential of AuNSs@GSH@ET‐7 only changed to −10.9 mV, due to the electrically neutral characteristic of ET‐7 molecule (Figure S39C, Supporting Information). All these results together demonstrated the successful assembly of AuNSs@GSH@ET‐R probes.

Then, the analytical performance of 7 molecules toward AchE was compared through Raman spectroscopy. As shown in Figure 1C, all the developed ET‐R molecules showed responses toward AchE. The red peaks (1325‐1360 cm^−1^) which were attributed to C─O stretching vibration were increased, while the green peaks ascribed to C≡C stretching vibration peak at 1970–2039 cm^−1^ remained unchanged. The selectivity tests of the developed AuNSs@GSH@ET‐R probes were first checked against potential biological species including amino acids, neurotransmitters, enzymes, metal ions, and reactive oxygen species (ROS) in the complex brain. As shown in Figure 1D, ET‐1 (80.6±1.4%), ET‐2 (32.8±2.0%) and ET‐3 (20.2±1.1%) demonstrated obvious responses toward BchE, while ET‐R (*R =* 4–7) showed negligible responses (< 6.83%) for other enzymes including butyrylcholinesterase (BchE), superoxide dismutase (SOD), monoamine oxidase (MAO), β‐site amyloid precursor protein cleaving enzyme (BACE1), choline acetyltransferase (CHAT) (200 mU mL^−1^), the common body‐contained esterase including carboxylesterase (CES), phosphodiesterase (PDE), phosphomonoesters (PMS), sulfatase (AS) and cyclic nucleotide phosphodiesterase (PDE), amino acids, neurotransmitters, and other biological species (Figure S40, Supporting Information). Furthermore, we found that the response time of ET‐7 (49 min) was significantly longer than those of ET‐R (*R =* 4–6). (Figure S41A, Supporting Information). More importantly, the Raman fluctuation of ET‐5 was increased by 124% with the shortest response time of 20 min, while those of ET‐4 and ET‐6 were only increased by 23% and 58% with the addition of 600 mU mL^−1^ AchE, respectively, indicating that ET‐5 showed the best sensitivity of Raman responses toward AchE (Figure S41B, Supporting Information). Accordingly, ET‐5 molecule was optimized as a promising candidate for in vivo monitoring of AchE levels because it showed the highest selectivity, the fastest response time, and the best Raman sensitivity.

Next, the titration of the optimized AuNSs@GSH@ET‐5 probe was performed toward the addition of AchE in cell lysis buffer or artificial cerebrospinal fluid (aCSF). Considering the strong tissue penetration and avoiding autofluorescence interference, a laser with an excitation wavelength of 785 nm was selected for the following SRES measurements. As shown in Figure 1E, a significant enhancement in Raman signals was observed for ET‐5 molecules assembled onto AuNS substrate. SERS enhancement factor (EF) was calculated to be 4.55 × 10^6^, in which 4‐Mercaptobenzoic acid was chosen as a model molecule (Figure S42, Supporting Information). With the addition of AchE, the peak intensity at 1350 cm^−1^ (I_1350_) was gradually increased with increasing activity of AchE from 0 to 500 mU mL^−1^, which was attributed to C─O stretching vibration. Meanwhile, C≡C stretching vibration peak (2031 cm^−1^) remained unchanged, which can be employed as an inner reference for providing built‐in corrections, thus improving the accuracy for quantitative determination of AchE (Figure 1E). As demonstrated in Figure 1F, SERS intensity ratio I_1350_ / I_2031_ showed good linearity with the activity of AchE in the range of 0.1–400.0 mU mL^−1^ with a sensitivity of (7.0±0.4) × 10^−3^ mL mU^−1^ (*n* = 10, S. E. M.). The detection limit was estimated to 49.5 ±3.2 µU mL^−1^ (S/N = 3, *n* = 10). More significantly, no obvious changes (< 4.72%) were observed for 12 TFs, proving good reproducibility (Figure S43, Supporting Information). All the results indicated that the developed AuNSs@GSH@ET‐5 probe can be used for selective and accurate quantification of the activity of AchE, which should be ascribed to the specific recognition as well as built‐in correction abilities of ET‐5 toward AchE.

### 2.2 The Recognition Mechanism of AuNSs@GSH@ET‐R Towards AchE

**Figure 2:**
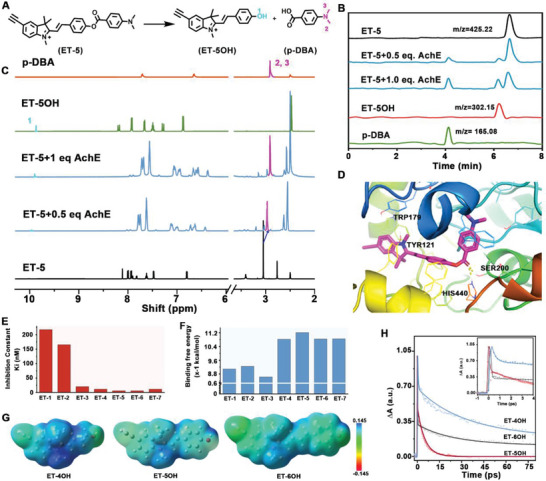
A) The principle of recognition mode of ET‐5 molecule for recognition of AchE. B and C) LC‐MS spectra B) and partial ^1^H NMR titration spectra C) of ET‐5 (5 mM) upon addition of AchE (0, 0.5, and 1 equiv.). D) Molecular docking simulation of ET‐5 to AchE. E and F) Summarized data of E) inhibition constant and F) binding free energy for ET‐R (*R* =1–7). G) Electron cloud density distribution of ET‐4OH, ET‐5OH and ET‐6OH. H) Transient absorption kinetics of ET‐ROH (*R* =4–6) with 600 mU mL^‐1^ AchE.

In order to understand the recognition mechanisms of the synthesized 7 molecules toward AchE, liquid chromatography−mass spectrometry (LC‐MS) and ^1^H NMR experiments were first used to confirm the reaction products after ET‐Rs were reacted with AchE (Figure S44, Supporting Information). After addition of AchE into ET‐R probes, two different products with m/z peaks were all observed for 7 molecules, which were attributed to 5‐ethynyl‐2‐(4‐hydroxystyryl)−1,3,3‐trimethyl‐3H‐indol‐1‐ium (ET‐ROH) and carboxylic acid derivatives (R─COOH), respectively, as demonstrated in **Figure** **2**A,B. Furthermore, ^1^H NMR was employed to check the changes of chemical groups before and after ET‐Rs were reacted with AchE. As shown in Figure 2C, two new peaks, which were attributed to H_1_ of ‐OH group in ET‐ROH and H_2,3′_ of ─CH_3_ in R─COOH, were observed, and these peak intensities were gradually increased with increasing activity of AchE (Figure S44B, Supporting Information). This observation indicated that addition of AchE resulted in the break of C─O bond in ET‐R (Figure 2A). All these results strongly demonstrated that the responses of the developed 7 molecules toward AchE were due to the break of ester bonds to generate ET‐ROH and R─COOH.

Next, molecular docking simulation was used to compare the cavity matching degree, binding free energy, and inhibition constant (*K*_0_) between ligand and AchE for evaluating the selectivity and response time of seven molecules toward AchE. As shown in Figure 2D, only ET‐2 and ET‐5 matched the AchE cavity and formed hydrogen bonding with AchE active hydrolysis sites Ser200 and His440 (Figure S45, Supporting Information). Meanwhile, as shown in Figure 2E,F, ET‐R (*R =* 4–7) presented obviously higher binding affinity (> −10.40 kcal mol^−1^) and lower inhibition constant (< 10 nM) toward AchE than ET‐R (*R =* 1–3). More importantly, ET‐5 showed the smallest inhibition constant (6.03 nM) and the highest binding energy (−11.42 kcal mol^−1^) among the designed molecules, suggesting the best selectivity of ET‐5 for recognition of AchE. The results agreed with those observed in the experimental data as shown in Figure 1D. In addition, it was found that the nitrogen cation of ET‐R (*R =* 4–6) could form electrostatic interaction with the peripheral anion sites of AchE Tyr121 and Trp279, to accelerate the binding process to the AchE active center, which was favored for promoting the response time toward AchE (Figure 2E).^[^ ^10^ ^]^ Thus, ET‐R (*R =* 4–6) demonstrated a faster response time than that of ET‐7, as proved in the experimental results (Figure S41A, Supporting Information).

Furthermore, the charge distributions were estimated by DFT calculation, including the electron static potential (ESP) and natural population analysis charges (NPA), to evaluate the response abilities of different conjugated molecules ET‐R (*R =* 4–6) toward AchE (Figure S46, Supporting Information).^[^ ^11^ ^]^ The Raman response‐ability is affected by the change in bond polarity, which is related to the change in the electron distribution. As shown in Figure 2F, the ESP distribution showed that the electron cloud of ET‐R (*R =* 4–6) was initially biased towards the ester group. After reacting with AchE, the charge distributions of the products of ET‐5OH and ET‐6OH were more delocalized than that of ET‐4OH, which indicated that the reactions of AchE with ET‐R (*R =* 5,6) caused the electron transfer on the carbonyl oxygen, and the generated phenolic salt ions had the electron‐donating effect, thus forming π‐conjugated systems with a “push‐pull” structure.^[^ ^12^ ^]^ Meanwhile, NPA charge calculation showed that the electronegativity of oxygen atom in ET‐5OH molecule (−0.641) was significantly lower than those in ET‐4OH (−0.748) and ET‐6OH (−0.648), demonstrating that the conjugated structure promoted the charge transfer of phenolic salt ions (Figure S46, Supporting Information). The extension of the conjugated molecule for ET‐6 with chain lengths longer than 2 led to weakening electron transport efficiency. The lower electronegativity of the bonding atoms increased the mobility of electrons, which in turn increased the polarizability of the bond.^[^ ^13^ ^]^ Furthermore, as shown in Figure 2G, new decay pathways were observed from transient absorption (TA) kinetic spectra of ET‐ROH (*R =* 4–6), which were attributed to intramolecular electron transfer state. The decay kinetics of ET‐5OH (0.06 ps) was obviously faster than those of ET‐4OH (8.26 ps) and ET‐6OH (0.34 ps), further demonstrating that the electron transfer efficiency of ET‐5 molecule was the highest among these 3 molecules. Therefore, the polarity changes of C─O in ET‐5 showed the largest Raman response sensitivity, consistent with the results obtained in Figure 1C. Accordingly, the ET‐5 molecule was optimized as a promising candidate for in vivo monitoring of AchE levels because it shows the highest selectivity, the fastest response time, and the best Raman sensitivity among the designed 7 molecules.

### 2.3 Determination and Regulating of AchE Activity in NSCs

**Figure 3:**
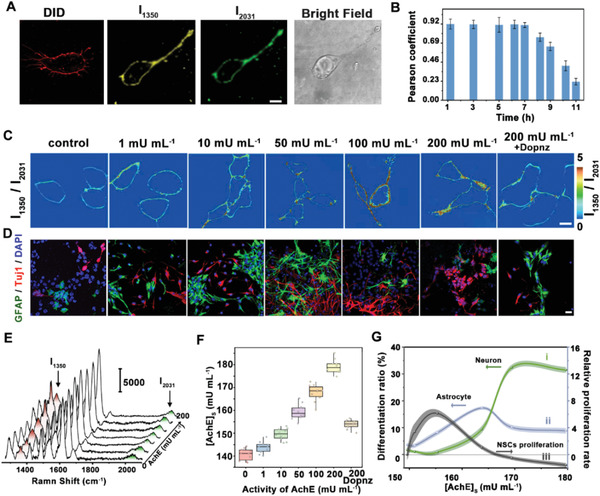
A) Fluorescence and Raman images of NSCs treated with DID and AuNSs@GSH@ET‐5. Scale bar: 15 µm. B) Pearson's correlation coefficient statistics between AuNSs@GSH@ET‐5 and DID on NSCs synapses for different times (1.5, 3, 5, 6.5, 7, 8.5, 9, 10.5, 11 h) (*n* =10, S. E. M.). C) The ratiometric SERS imaging of I_1350_/I_2031_ obtained from NSCs which were incubated with different activities of AchE (0, 1, 10, 50, 100, 200 mU mL^‐1^, and 200 mU mL^‐1^ AchE+10 µM donepezil), scale bar: 10 µm. D) Immunofluorescence imaging of Tuj1 and GFAP after NSCs were incubated with different activities of AchE (0, 1, 10, 50, 100, 200 mU mL^‐1^, and 200 mU mL^‐1^ AchE+10 µM donepezil), scale bar: 20 µm. E) The Raman spectra of AuNSs@GSH@ET‐5 were obtained from NSCs in C). F) The AchE activity on synapse after the NSCs was stimulated by different activities of AchE (0, 1, 10, 50, 100, 200 mU mL^‐1^, and 200 mU mL^‐1^ AchE+10 µM donepezil). G) The relative proliferation and differentiation rates of NSCs with different AchE activities of synapse (148.37, 152.06, 155.89, 167.39, 169.91, 179.52 mU mL^‐1^). (*n* =10, S. E. M.)

For further applications in cells and brains, the stability of AuNSs@GSH@ET‐5 probe was estimated. Raman imaging results demonstrated that the AuNSs@GSH@ET‐5 probe was the same location in NSCs with commercial probe, 1,1′‐Dioctadecyl‐3,3,3′,3′‐tetramethylindodicarbocyanine perchlorate (DID), which exhibits a marked increase in fluorescence when bound to membranes (**Figure** **3**A; Figure S47A, Supporting Information), suggesting that the developed probe was mainly targeted and located in synapse. In addition, no obvious change (< 3.24%) was obtained after AuNSs@GSH@ET‐5 was anchored onto the synapse for 7 h (Figure 3B; Figure S47A, Supporting Information). Then, pharmacokinetic fluorescence imaging of brain slides and organs demonstrated that most of the AuNSs@GSH@ET‐5 probes (> 96.03%) were metabolized through kidney and liver after being injected into brain for ≈40 h (Figure S47B,C, Supporting Information). Next, the biosafety of AuNSs@GSH@ET‐5 probe was also evaluated. MTT results showed that the cell viability was higher than 90.65±1.32% after the nanoprobe (15.6 µg mL^−1^) was incubated with NSCs for 48 h (Figure S48A, Supporting Information). In addition, the results of flow cytometry sorting (FACS) confirmed that there was no significant difference in living cells, apoptotic cells, and dead cells after the cells were incubated with the nanoprobes even with a high concentration of 15.6 µg mL^−1^ (Figure S48B, Supporting Information). In addition, due to the biocompatible of AuNSs, the probe could circulate metabolically through blood vessels and does not induce any immune reactions.^[^ ^14^ ^]^ All these results demonstrated that the developed AuNSs@GSH@ET‐5 probe showed high stability, low toxicity, and good biocompatibility, which was very beneficial for imaging and quantifying AchE in live cells and brains.

Then, the developed nanoprobe was applied for monitoring the activity of AchE released from NSC synapses. NSCs, important neural cells from the central nervous system, have the potential to differentiate into neurons and astrocytes and play key roles in the reconstruction and functional recovery of neural pathways.^[^ ^15^ ^]^ Under physiological conditions, the activity of AchE on NSCs synapse was estimated to be 143.11±7.60 mU mL^−1^ (*n* = 10, S. E. M.). With increasing extracellular AchE activity from 1–200 mU mL^−1^, Raman intensity of *I*_1350_/*I*_2031_ was increased. The activity of AchE was increased from 143.11±7.60 to 183.32±11.44 mU mL^−1^ (*n* = 10, S. E. M. Figure 3C,E,F). In order to further explore the effect of AchE on the differentiation ability of NSCs, immunofluorescent detection of the astrocyte marker glial fibrillary acidic protein (GFAP) and the neuronal marker neuronal class III β‐Tubulin (Tuj1) were performed after NSCs were stimulated by different activities of AchE (1–200 mU mL^−1^ Figure 3D,G; Figure S49A, Supporting Information). Flow cytometry showed that the differentiation rate of NSCs was 5.74±0.85% without stimulation by AchE, in which the differentiated rate of astrocytes was 3.64±0.64%, and that of neurons was 1.74±0.57%. More interestingly, as demonstrated in Figure 3G, with increasing activity of AchE, the differentiation rate of NSCs was significantly increased. After AchE activity in NSC synapse was increased to 163.71 mU mL^−1^, the differentiation rates of NSCs into astrocytes reached the highest value of 19.76±1.53%, and that of neurons was 8.32±0.87% (Figure S49A, Supporting Information). Then, the differentiation rate of astrocytes gradually decreased, while that of neurons continued to increase with the following increase in activity of AchE. After AchE activity was stimulated to 172.40 mU mL^−1^, the differentiation rate of neurons showed the highest value of 34.29±4.29%; while that of astrocytes decreased to 11.47±2.63%. Furthermore, confocal fluorescence imaging shown in Figure 3D confirmed that the results were consistent with those obtained by flow cytometry. Furthermore, after adding donepezil (Dopnz, acetylcholinesterase inhibitor), the activity of AchE on the synapse was reduced to 154.37 ± 9.11 mU mL^−1^ (*n* = 10, S. E. M.), and the differentiation rate of NSCs was weakened (9.94±2.27%) (Figure 3C,E,F). The result further confirmed the ability of AchE to regulate NSCs differentiation. All those results proved that the increased activity of AchE can regulate the differentiation of NSCs into neurons and astrocytes.

More interestingly, AchE was also found to regulate the proliferation of NSCs. According to flow cytometry, AchE activity on the cell membrane of NSCs had an obvious proliferative effect with an optimized value from 149.53 to 167.39 mU mL^−1^. The best proliferative effect of 6.52±0.71% was observed with the optimized activity of AchE,155.89±6.26 mU mL^−1^. However, after the AchE activity was higher than 167.39 mU mL^−1^, the proliferation of NSCs was inhibited instead (Figure 3G; Figure S49B, Supporting Information). All these results indicated that the optimized AchE activity could regulate both proliferation and differentiation of NSCs, which has never been reported. This discovery should be attributed to quantitative determination of AchE with high accuracy by our developed method.

### 2.4 Raman Photometry Array With the Tapered Fibers for Accurate Determination AchE In Vivo

**Figure 4:**
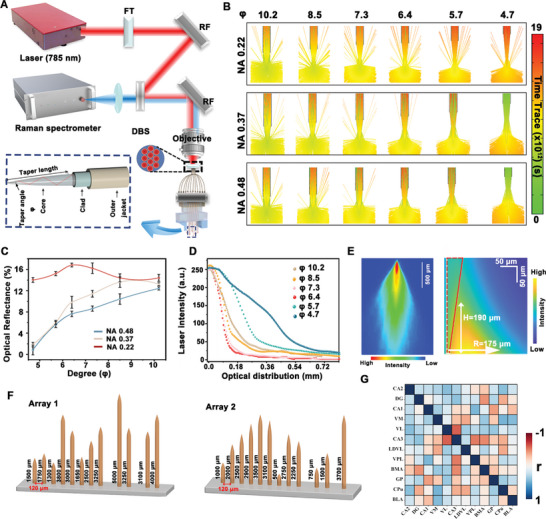
A) Optical setup of hign‐density Raman fiber photometry. FT, RF, and DBS represent filter, reflector, and dichroic beam splitter, respectively. B) Ray‐tracing simulations of emissions from the TFs (NA 0.22, 0.37, and 0.48) resulting from injecting a single ray into the fiber at different angles (*φ*=4.7°, 5.7°, 6.4°, 7.3°, 8.5° and 10.2°). C) The optical transmittance of TFs (NA 0.22, 0.37, and 0.48) with different angles (*φ*=4.7°, 5.7°, 6.4°, 7.3°, 8.5°, and 10.2°) obtained from B). D) The emission length of TFs (NA 0.22) with the specified taper angles (*φ*=4.7°, 5.7°, 6.4°, 7.3°, 8.5°, and 10.2°). E) Experimental and theoretical simulations of the optical field distribution of the tapered fiber tip. F) Schematic diagram of the developed multi‐fiber microarray. Array 1 at 1.98 mm posterior bregma; Array 2 at −1.22 mm posterior bregma. G) The measurement of signal cross‐talk between fiber channels. (*n* =10, S. E. M.)

In order to record and study the functional signals of neural populations in multiple brain regions of freely moving animals, herein, a fiber array with 12 fibers was constructed for simultaneously collecting the signals from multiple brain regions using multi‐fiber Raman photometry. As shown in **Figure** **4**A, twelve multimode fibers (100 µm core/25 µm cladding) were tightly bundled together at one end and separated independently at the other end. The excitation light (785 nm) was first delivered into the confocal scanner, then coupled to the bundled end of optical fiber by an objective (10×), further exciting the Raman probes at another end of fiber. Then, Raman signals of the probes were collected with the same optical fiber and then detected by a Raman spectrometer. The excitation light was coupled into different fibers individually through the confocal scanner.

The tapered fibers (TFs) were used in the array because of minor invasiveness and larger light collection volume. To both maximize the signal collection efficiency of the fiber and avoid signal crosstalk between adjacent brain regions, we optimized the numerical aperture (NA) and taper angle (*φ*) of the fiber. Ray‐tracing and geometric models demonstrated the working principle of the TFs. A ray injected into the core of the fiber is guided to the tapered region via total internal reflection. After the reflected ray meets a critical section, total internal reflection is lost and the ray radiates into the surrounding medium (Figure 4A). The ray tracing is based on the Helmholtz equation (Equation 1) to design and simulate the performance of TF, which is used to accurately analyze the propagation process of light.^[^ ^16^ ^]^ The tip diameter of TF was fixed as 10 µm, and the taper angle φ (4.7–10.2) was regulated by the taper length *L* (0.25‐0.5 mm) according to Equation 2. The materials forming all the components of the TFs and the surrounding media were assumed to be homogenous (refractive index constant in space). As shown in Figure 4B, through modeling and simulation of TF (φ = 4.7°, 5.7°, 6.4°, 7.3°, 8.5° and 10.2°) with different NAs (0.22, 0.37, 0.48), it is found that TF with NA 0.22 and taper angle of 6.4 demonstrated the maximum optical reflectance (16.3%) (Figure 4B,C).

(1) $$\left(\nabla^{2}+k^{2}\right)A=0$$

where ∇ is Hamiltonian, *k* is wave number and *A* is amplitude.

(2) $$\varphi =arctg\frac{R-r}{L}$$

where *R* is the core radius, *r* is the taper radius and *L* is the taper length.

Furthermore, the simulation results were verified by experiments. TFs (NA 0.22) with different taper angles were fabricated by a CO_2_‐laser‐based micropipette puller. SEM image showed that the tip diameter of TFs was 10 µm, and the taper angles were from 4.7°, 5.7°, 6.4°, 7.3°, 8.5° to 10.2° (Figure S50, Supporting Information). Then, TFs were immersed in a fluorescein solution, and imaging the resulting optical distribution. As shown in Figure 4D, by comparing the emission lengths of different TFs, it was found that the length of the emitting segment increased with decreasing the taper angle. The taper angle of 6.4° demonstrated the maximum emission length (100 µm), which was consistent with the modeling (Figure 4E). Meanwhile, the simulation of the optical field distribution showed that the collection field of optimized TFs (NA 0.22, φ 6.4°) was 6.25 × 10^−3^ mm^3^, which was consistent with the light collection characteristics of TFs in fluorescent solution (Figure 4E). Therefore, based on the optimized results, two microarrays with 12 TFs were designed as shown in Figure 4F, with different lengths and adjacent distances (120 µm) to match the depths of brain regions. Finally, we verified that there was no signal cross‐talk between fiber channels (Figure 4G).

### 2.5 Tracking of AchE in the brain of freely moving AD mice with network

**Figure 5:**
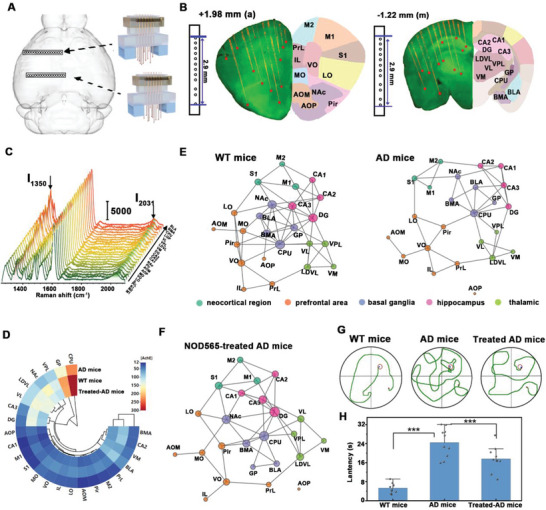
A) The top view of brain surface with implanted multi‐fiber arrays (12‐fiber arrays at 1.98 mm anterior and 1.22 mm posterior bregma, respectively). B) 3D reconfiguration Mapping aligned to Allen Brain Atlas. Fiber tracks in yellow with fiber tip positions (red dots) mapped to corresponding brain regions. The fiber array distributed across areas in anterior cortex (a), medial areas of the basal ganglia–thalamocortical loop (m). C) Raman spectra of AuNSs@GSH@ET‐5 probe in 24 brain regions collected from 20 weeks WT mice. D) The activities of AchE in 24 brain regions collected from WT mice, AD mice, and 50 µM NOD565‐treated AD mice. E) Brain network plot of 24 brain regions collected from WT mice and AD mice. Region nodes are colored according to their number of links. Note that a higher density of links affects the circle areas. F) Brain network plot of 24 brain regions collected from NOD565‐treated AD mice. Region nodes are colored according to their number of links. Note that a higher density of links affects the circle areas. G) The escape tracking trace in MWM with a platform of the WT mice, AD mice, and 50 µM NOD565‐treated AD mice. H) Escape latency to reach the safety platform of different treatment AD mice.(*n* =10, S. E. M. *, *p* < 0.05; **, *p* < 0.01; ***, *p* < 0.001)

Next, 24 fibers of two 12‐fiber microarrays were implanted into two mice and distributed across two front pieces in one hemisphere (**Figure** **5**A). This approach allowed us to quantify the activities of AchE from a large‐scale network distributed across anterior cortex (neocortical regions M1, M2, S1 and prefrontal areas PrL, IL, DP, MO, LO, AOM, AOP, Pir), hippocampus areas CA1, CA2, CA3, DG, basal ganglia CPu, BLA, BMA, GP, NAc, and thalamic VL, VM, VPL, LDVL. All fiber arrays pre‐adsorbed with the fluorescent indicator Cy3 were inserted into the brains of EGFP mice. The three‐dimensional (3D) location of the fiber tips and the neuronal labeling in front of these fiber tips in the brain by 3D reconstruction demonstrated accurate localization of the developed fiber arrays into the target brain regions (Figure 5B, Figure S51A, Supporting Information). Meanwhile, the fiber showed no significant trauma and tissue immune response after microarray was implanted into the brain for 3 h (Figure S51B,C, Supporting Information), demonstrating the good biocompatibility of the developed microarray.

Then, the distribution of AchE activities in 24 brain regions of C57/BL‐6 wild type (WT) mice and APPswe / PS1dE9 double transgenic AD mice (2×Tg‐AD, 20 weeks old) were first monitored. As shown in Figure 5C,D, the distribution of AchE activity varied in different brain regions: the highest AchE activity of 318 ± 12 mU mL^−1^ was observed in CPu region, which was 6 times greater than that in anterior cortex regions (51 ± 4 mU mL^−1^). Interestingly, AchE activity in CA1 region (23 ± 5 mU mL^−1^) was significantly lower compared with those in hippocampal DG and CA3 regions (146 ± 11 mU mL^−1^). For AD mice, the activity of AchE in the hippocampus (CA1, CA2, CA3, DG) reduced by 32 ± 3%, those in cortex and amygdala (BMA, BLA) decreased by 17±5% and 22±5% (*n* = 10, S. E. M., Figure S52, Supporting Information), respectively. No obvious changes were observed for other brain regions. It suggested that the reduction of AchE activity may be closely related to AD induction.

Furthermore, the microarrays were used to reconstruct the functional networks of adjacent brain regions related to AchE. Pairwise correlations between AchE activity of different brain regions were calculated, in which significant correlations were connected via links.^[^ ^17^ ^]^ With 24 brain areas modeled in this study, this investigation offered 276 cross‐correlations to test. A schematic representation summarizing the main correlations network between brain regions was summarized in Figure 5E. It was found that the brain network formed several different clusters (neocortical regions M1, M2, S1, prefrontal areas PrL, IL, DP, MO, LO, AOM, AOP, Pir, hippocampus areas CA1, CA2, CA3, DG, basal ganglia CPu, BLA, BMA, GP, NAc, and thalamic VL, VM, VPL, LDVL). As for WT mice, there were 60 significant cross‐correlations, in which CPU, NAc, CA3, and DG regions showed more connections (> 8) than other regions, and occupied the central network position. However, for AD mouse, the cross‐correlation of 24 brain regions were significantly attenuated to 40, where the connection of NAc, CA3, and DG regions decreased to less than 5. The correlation between hippocampal and cortical neuroclusters significantly decreased, in which the association between prefrontal cortex and hippocampus was lost, and the central network position was decreased to CPu‐the mainly cholinergic system.^[^ ^18^ ^]^ Prefrontal cortex‐hippocampus circuit was reported as a neural circuit closely related to spatial cognition.^[^ ^19^ ^]^

The built‐fiber Raman device not only established the approach for in vivo monitoring of AchE activities in multiple brain regions but also provided in situ photo‐modulated sources in the deep brain by coupling different wavelengths of light. Nitric oxide (NO) is an important neurotransmitter in the brain and is closely related to treating AD by regulating neuronal activity. Recent studies have shown that there is extensive interaction between the novel neurotransmitter NO and the cholinergic system. The cholinergic system can act by influencing the NO system.^[^ ^20^ ^]^ In order to achieve controllable regulation in vivo, NO donor NOD565 was introduced to quantitatively release NO.^[^ ^21^ ^]^ NOD565 was injected into hippocampus and CPu regions for AD mice. As shown in Figure 5D, after photoactivated NOD565 for 30 s, the activity of AchE in the hippocampus (CA1, CA2, CA3, DG) was recovered by 12 ± 5%, and that in CPu was increased by 22.5± 7.38%. Meanwhile, the cross‐correlation of 24 brain regions related to AchE was significantly enhanced from 40 to 47 (Figure 5F). The correlation between hippocampus and cortical neural clusters was found to be recovered. Furthermore, Morris water maze (MWM) was used to evaluate the cognitive function of AD mice. It was found that photo‐activated NOD565 improved the cognition recovery of AD mice, the latency was decreased to 17±4 s compared with AD mice (24±8 s) (Figure 5G,H). All these results demonstrated that the loss of AchE functional network in AD mice was reconstructed by the controlled release of AchE regulated by NO, which was closely related to the treatment of AD.

## 3 Conclusion

We have designed and synthesized a series of ET‐R derivatives for specific recognition of AchE. By rationally tuning the conjugation and substitute groups of the designed organic molecules, ET‐5 was optimized for an effective Raman biosensor based on a dual‐recognition strategy to specifically recognize AchE in the brains of free‐moving animals. Meanwhile, a Raman fiber photometry with 24 TFs was built for recording AchE activity in 24 brain regions across the whole brain of freely moving animals. We found that the activity of AchE varied in 24 brain regions, which was significantly decreased in AD mice compared with that in WT mice. By using our probe and high‐density Raman fiber photometry, the AchE functional network of 24 brain regions was established for the first time. Meanwhile, we found that the loss of AchE functional network in AD mice could be restored and reconstructed by the controlled quantitative release of AchE by NO.

Our work has provided a methodology for designing and optimizing the efficient Raman biosensor for enzymes and proteins with high selectivity, accuracy, and sensitivity. Meanwhile, using the developed high‐density fiber Raman platform, we have established functional networks in multiple brain regions of the living brain. Furthermore, our method can be correlated with behavioral and disease models of freely moving animals to evaluate the functional relationship between biologically active substances and animal behaviors, thereby realizing the analysis of physiological and pathological mechanisms and the screening of drugs.

## 4 Experimental Section

### Cytotoxicity, Apoptosis, and Biosafety Assays

For cytotoxicity assay, AuNSs@GSH@ET‐5 nanoprobes with different concentrations (0, 3.1, 6.2, 9.4, 12.6, and 15.6 µg mL^−1^) were added to the 96‐well plates pre‐incubated with neural stem cells and further cultured for 24 and 48 h, respectively. Subsequently, 20 uL Thiazolyl blue (MTT) was added to each well. After 4 h of reaction, the mixed solution was removed and 80 µL dimethylsulfoxide (DMSO) was added. Then, after shaking the cells for 5 min, the absorbance at 490 nm was measured. According to the following formula to determine cell viability: cell viability (%) = experimental group absorbance / blank control group absorbance × 100%.

For the determination of apoptosis, different concentrations of AuNSs@GSH@ET‐5 nanoprobes (3.1, 9.4, 12.6, and 15.6 µg mL^−1^) were cultured with neural stem cells for 24 h. Then the cells were digested and re‐suspended in 300 µL binding buffer and further incubated with FITC‐annexin V and propidium iodide (PI) in a dark environment for 30 min. Apoptotic cells and necrotic cells were labeled respectively, and apoptosis was measured by flow cytometry.

In order to evaluate the biological safety of the developed probe, AuNSs@GSH@ET‐5 (5 µL, 15.6 µg mL^−1^) was first injected into the mouse brain through a micro syringe (Hamilton), and then the mouse brain was quickly removed at different time intervals. Meanwhile, different organs (heart, liver, spleen, lung, and kidney) of the mouse were removed for in vivo imaging.

### Calculation of *EF*

The *EF* for each nanomaterial was calculated by the following Equation (3).^[^ ^22^ ^]^

(3) $$EF=\left(I_{\mathrm{SERS}}/N_{\mathrm{ads}}\right)/\left(Ib_{\mathrm{ulk}}/N_{\mathrm{bulk}}\right)$$

*N*_ads_ and *N*_bulk_ represented the number of 4‐Mercaptobenzoic acid (4‐MBA) molecules in the SERS sample and the normal Raman sample, respectively. *I*_SERS_ and *I*_bulk_ were the same vibration peak of the 4‐MBA molecule on one AuNSs and the normal Raman spectrum from the solid sample, respectively. *I*_SERS_ and *I*_bulk_ were obtained on the peak intensity of 4‐MBA molecule at 1077 cm^−1^ on AuNSs substrates (Supplementary Figure S17). The average intensity of 4‐MBA at 1077 cm^−1^ (*I*_SERS_ = 8500 and *I*_bulk_ = 150) was used for the calculation of the *EF* value.

In the experiment, 100 µL aqueous 4‐MBA solution (10 mM) was dried onto the Si wafer (0.5 cm by 0.5 cm), and *N*_bulk_ can be estimated as:

(4) $$N_{\mathrm{bulk}}=100\;\mu L\times 10^{-2}\;M\times 6.02\times 10^{23}{\mathrm{mol}}^{-1}\times 4.50\;{\mu m}^{2}/0.25\;{\mathrm{cm}}^{2}$$

where *d* is the diameter of the light spot (d = 1.22λ / NA), *λ* is the incident wavelength (785 nm), and NA of the objective lens is 0.4, thus the laser spot size [π(d/2)^2^] is ≈4.50 mm^2^. *N*_bulk_ was estimated to be 1.083 × 10^11^.

(5) $$N_{\mathrm{ads}}=0.5\;\mathrm{nM}/{\mathrm{cm}}^{2}\times 6.02\times 10^{23}{\mathrm{mol}}^{-1}\times 4.50\;{\mu m}^{2}=1.35\times 10^{6}$$

Thus, the *EF* of AuNSs was calculated to be (4.55 ± 0.46) ×10^6^ (*n* = 5, S. D.)

### Molecular Docking Simulation

The molecular structure ET‐5 was drawn by ChemDraw ChemBioDraw Ultra 14.0, and the structure was imported into ChemBio3D Ultra 14.0 for energy minimization. The minimum RMS Gradient was set to 0.001. Import the optimized molecules into AutodockTools‐1.5.6 for hydrogenation, calculation of charge, and distribution of charge. According to the protein (PDB ID: 1EVE), the protein structure was downloaded from the PDB database, and the crystal water of the protein was removed. Import the processed protein structure into AutoDock tools (v1.5.6) for hydrogenation, calculation of charge, distribution of charge, and specifying atomic type. The relevant parameters were center _ *x* = 1.057, center _ *y* = 60.056, and center _ *z* = 71.240; the lattice box size was set to 80 × 80 × 80 (the spacing of each lattice point was 0.375Ω), and its parameters were default. PyMOL2.3.0 and LigplotV2.2.4 were used to analyze the interaction mode of docking results.

### Molecular Dynamics Simulation

Gromacs 2020.3 software was used to simulate the molecular dynamics of the system. The complex was placed in a twelve‐square box and TIP3P water was added. The system was neutralized with Na^+^ and Cl^−^, and the concentration of NaCl solution was set at 0.15 M. First, the maximum energy of the system is less than 1000 kJ mol^−1^ nm^−1^ by minimizing the energy. Then, the system was balanced in two steps, so that the temperature of the system is stabilized at 300 K and the pressure was stabilized at 1 bar. Finally, the dynamic simulation was carried out for 100 ns. Furthermore, the binding free energies of complexes formed by ET‐R and proteins were calculated.

### Design and Built of High‐Density Raman Fiber Photometer

Raman fiber photometer was used to record Raman signals. 12 multimode fibers (100 µm core/30 µm cladding) were tightly bundled together at one end and separated independently at the other end. The excitation light was generated by the fiber laser (Laser‐785‐5HS, Shanghai Ruhai Optoelectronics) and delivered into the confocal scanner (Scan‐Vis‐G, Thorlabs), and then coupled through the objective lens (10× / NA 0.25, Leica) to further excite the fibers. Then, the Raman signal of the probe was collected with the same fiber and detected with a Raman spectrometer (QE65 Pro, Ocean Optics). The excitation light was coupled to different fibers by confocal scanner (Scan‐Vis‐G, Thorlabs), and the signal was collected one by one. The signal acquisition time interval between adjacent fibers was 5 s. In addition, the end of each split fiber was tapered with a laser drawing machine (P‐2000 Laser‐Based Micropipette Puller, Sutter) to optimize the signal acquisition efficiency, and the diameter of the optimized fiber tapered tip was 10 µm. To prevent interference from excitation light, filters (LPD02‐785RU, Semrock) and dichroic beam splitters (NF03‐785E‐25, Semrock) were used in this system. Then, 24 fibers of two 12‐fiber microarrays were implanted into two mice for detecting the AchE activity by the developed system.

### Immunofluorescence Imaging

NSCs were treated with different activities of AchE, after that, the mixture was washed five times with PBS, and the cells were fixed with 4% paraformaldehyde at 37 °C for 30 min. Then, the samples were washed with PBS, and 2% PBST was added for 15 min of cell membrane permeability. After washing with PBS, the sample was added to 10% goat serum and sealed at 25 °C for 1 h. Next, the sample was further washed three times with PBS and added anti‐GFAP antibody (abcam, ab7260) and anti‐Tuj1 antibody (abcam, ab14545) (1: 200) and incubated at 4 °C overnight. After that, the sample was washed with PBS. Then Cy3‐AffiniPure Goat Anti‐Mouse IgG (H+L) (Yeasen, 33208ES60) and FITC‐AffiniPure Goat Anti‐Rabbit IgG (H+L) (Yeasen, 33107ES60) (1: 500) were added and incubated at 37 °C for 1 h. After staining, the samples were washed with PBS for three times. Then, DAPI solution and dye were added for incubation for 5 min. After washing with PBS for three times, the samples were analyzed by confocal fluorescence imaging and flow cytometry.

### In Vivo Raman Measurements

All animal experiments were performed according to the guidelines of the Care and Use of Laboratory Animals formulated by the Ministry of Science and Technology of China and were approved by the Animal Care and Use Committee of East China Normal University (approval no. m+ R20190304, Shanghai, China). In order to realize the detection of AchE activity in different brain regions of C57/BL6 mice or AD mice (20 weeks old), mice were anesthetized with 2% isoflurane, and the temperature of mice was maintained at 37 °C. Then, the mouse head was fixed on the stereo director. Hydrogen peroxide was applied to the skull surface to remove connective tissue allowing observation of the anterior fontanelle along the midline. Subsequently, according to the standard stereotaxic program, a rectangular hole was drilled in the upper part of the brain, which can span 12 brain regions. Then, the developed fiber array was fixed in the rectangular hole of mouse brain through dental cement. The anesthesia system was then removed to wake up the mice and adapt for 3 days.

In order to monitor the change of AchE activity in different brain regions of free‐living mice stimulated by NOD565, the mice were anesthetized with 2% isoflurane, and the temperature was maintained at 37 °C. According to the standard stereotaxic program, AuNSs@GSH@ET‐5 (5 µL, 15 .6 µg mL^−1^) was slowly injected into 12 brain regions of interest by micro syringe (Hamilton), and 5 µL 50 µM NOD565 was slowly injected into SGZ region. About 20 min later, the microarray was implanted into the brain and fixed with dental cement to prevent its shedding. Next, remove the anesthesia system to wake up the mice. A 532 nm laser was applied to activate NOD565 for 30 s in the hippocampus and CPu regions, and the AchE activity in each brain area within 3 h was continuously monitored after releasing NO.

### Statistical Analysis

Data were expressed as means ± S. E. M. of 5–10 samples in each experimental group. Statistical significance was determined by two‐sided Welch's ANOVA. The values 0.05 (*), 0.01 (**), and 0.001 (***) were assumed as the level of significance for the statistic tests (*, *p* < 0.05; **, *p* < 0.01; ***, *p* < 0.001.)

The qualitative analysis of AchE corresponded to multiple correlative analyses using Pearson's correlation coefficient with corrections^[^ ^23^ ^]^ and drawing by chip lot. These analyses were performed for AchE for all of the 20 investigated mice. The correlations were separately performed in mice of WT mice, 20‐week AD mice, and NOD565‐treated AD mice (20‐week) to estimate AchE connectivity in each group with no attempt to statistically compare the profiles. As previously reported,^[^ ^24^ ^]^ *p*‐values were adjusted using the False Discovering Rate (FDR) controlling procedures. Correlations were then considered significant at the 5% level.

## Conflict of Interest

The authors declare no conflict of interest.

## Supporting information

Supporting Information

## Data Availability

The data that support the findings of this study are available from the corresponding author upon reasonable request.

## References

[1] a) H. Soreq , S. Seidman , Nat. Rev. Neurosci. 2001, 2, 294; 11283752 10.1038/35067589

[2] a) L. Mucke , Nature 2009, 461, 895; 19829367 10.1038/461895a

[3] a) M. Behra , X. Cousin , C. Bertrand , J. L. Vonesch , D. Biellmann , A. Chatonnet , U. Strähle , Nat. Neurosci. 2002, 5, 111; 11753420 10.1038/nn788

[4] a) M. Grifman , N. Galyam , S. Seidman , H. Soreq , Proc. Natl. Acad. Sci. USA 1998, 95, 13935; 9811904 10.1073/pnas.95.23.13935 PMC24973

[5] a) J. y. Han , L. M. Besser , C. Xiong , W. A. Kukull , J. C. Morris , Alzheimer. Dis. Assoc. Disord. 2019, 33, 87; 30633043 10.1097/WAD.0000000000000291 PMC6542289

[6] a) F. Feng , Y. Tang , S. Wang , Y. Li , D. Zhu , Angew. Chem., Int. Ed. 2007, 46, 7882; 10.1002/anie.200701724 17768745

[7] a) J. Li , Y. Zhang , S. Ding , R. Panneerselvam , Z. Tian , Chem. Rev. 2017, 117, 5002; 28271881 10.1021/acs.chemrev.6b00596

[8] a) J. Liu , Z. Liu , W. Wang , Y. Tian , Angew. Chem., Int. Ed. 2021, 60, 21351; 10.1002/anie.202106193 34228388

[9] Z. Liu , Z. Zhang , Y. Liu , Y. Mei , E. Feng , Y. Liu , T. Zheng , J. Chen , S. Zhang , Y. Tian , Angew. Chem., Int. Ed. 2022, 61, 202111630. 10.1002/anie.202111630 35224847

[10] A. Hirashima , E. Kuwano , M. Eto , Bioorg. Med. Chem. 2000, 8, 653. 10732982 10.1016/s0968-0896(99)00315-6

[11] M. Zhu , L. Zhao , Q. Ran , Y. Zhang , R. Peng , G. Lu , X. Jia , D. Chao , C. Wang , Adv. Sci. 2022, 9, 2103896. 10.1002/advs.202103896 PMC8811804 34914857

[12] a) L. D. Patsenker , Y. Y. Artyukhova , J. Mol. Struct. 2003, 655, 311;

[13] a) J. Sopková , I. Císarová , Z. Arnold , Acta. Crystallogr. C 1996, 52, 2903;

[14] N. J. Abbott , A. A. Patabendige , D. E. Dolman , S. R. Yusof , D. J. Begley , Neurobiol. Dis. 2010, 37, 13. 19664713 10.1016/j.nbd.2009.07.030

[15] a) X. Li , X. Xie , Z. Ma , Q. Li , L. Liu , X. Hu , C. Liu , B. Li , H. Wang , N. Chen , C. Fan , H. Song , Adv. Mater. 2018, 30, 1804861; 10.1002/adma.201804861 30276898

[16] a) F. Pisanello , G. Mandelbaum , M. Pisanello , L. A. Oldenburg , L. Sileo , J. E. Markowitz , R. E. Peterson , A. Patria , T. M. Haynes , M. S. Emara , B. Spagnolo , S. R. Datta , M. D. Vittorio , B. L. Sabatini , Nat. Neurosci. 2017, 20, 1180; 28628101 10.1038/nn.4591 PMC5533215

[17] a) Y. Sych , M. Chernysheva , L. T. Sumanovski , F. Helmchen , Nat. Methods 2019, 16, 553; 31086339 10.1038/s41592-019-0400-4

[18] A. R. Helseth , R. Hernandez‐Martinez , V. L. Hall , M. L. Oliver , B. D. Turner , Z. F. Caffall , J. E. Rittiner , M. K. Shipman , C. S. King , V. Gradinaru , C. Gerfen , Science 2021, 372, eabe1931. 33888613 10.1126/science.abe1931 PMC8457366

[19] G. W. Wang , J. X. Cai , Behav. Brain Res. 2006, 175, 329. 17045348 10.1016/j.bbr.2006.09.002

[20] a) K. A. Petrov , A. I. Malomouzh , I. V. Kovyazina , E. Krejci , A. D. Nikitashina , S. E. Proskurina , V. V. Zobov , E. E. Nikolsky , Eur. J. Neurosci. 2013, 37, 181; 23121214 10.1111/ejn.12029

[21] H. He , Y. Xia , Y. Qi , H. Wang , Z. Wang , J. Bao , Z. Zhang , F. Wu , H. Wang , D. Chen , D. Yang , X. Liang , J. Chen , S. Zhou , X. Liang , X. Qian , Y. Yang , Bioconjug. Chem. 2018, 29, 1194. 29498825 10.1021/acs.bioconjchem.7b00821

[22] E. Feng , T. Zheng , X. He , J. Chen , Y. Tian , Sci. Adv. 2018, 4, eaau3494. 30406203 10.1126/sciadv.aau3494 PMC6214639

[23] C. K. Kim , S. J. Yang , N. Pichamoorthy , N. P. Young , I. Kauvar , J. H. Jennings , T. N. Lerner , A. Berndt , S. Y. Lee , C. Ramakrishnan , T. J. Davidson , M. Inoue , H. Bito , K. Deisseroth , Nat. Methods 2016, 13, 325. 26878381 10.1038/nmeth.3770 PMC5717315

[24] A. Fitoussi , F. Dellu‐Hagedorn , P. De Deurwaerdère , Neuroscience 2013, 255, 233. 24120557 10.1016/j.neuroscience.2013.09.059

