# Supplementary material for: A High‐Density Raman Photometry for Tracking and Quantifying of AchE Activity in The Brain of Freely Moving Animals with Network
Source: Adv Sci (Weinh). 2023 Aug 27;10(29):2301004. doi: 10.1002/advs.202301004 (PMC10582456; doi:10.1002/advs.202301004)
Supplement: Supplementary file 1 — Supporting Information [file ADVS-10-2301004-s001.pdf]

ADVANCED SCIENCE
Open Access

Supporting Information

for *Adv. Sci.*, DOI 10.1002/advs.202301004

A High-Density Raman Photometry for Tracking and Quantifying of AchE Activity in The Brain of Freely Moving Animals with Network

Zhonghui Zhang, Zhichao Liu, Peicong Wu, Xinhua Guo*, Xiao Luo, Youjun Yang, Jinquan Chen and Yang Tian**

Supplementary Information

A high-density Raman photometry for tracking and quantifying of AchE activity in the brain of freely moving animals with network

Zhonghui Zhang [1], Zhichao Liu [1], Peicong Wu [2], Xinhua Guo*[3], Xiao Luo [1], Youjun Yang [4], Jinquan Chen [2], and Yang Tian*[1, 2].*

[1] Dr. Z. Zhang, Dr. Z. Liu, Dr. X. Luo, Prof. Y. Tian
Shanghai Key Laboratory of Green Chemistry and Chemical Processes, School of Chemistry and Molecular Engineering
East China Normal University
Dongchuan Road 500, Shanghai 200241 (China)
E-mail: ytian@chem.ecnu.edu.cn

[2] Dr. P. Wu, Prof. J. Chen, Prof. Y. Tian
State Key Laboratory of Precision Spectroscopy
East China Normal University
Dongchuan Road 500, Shanghai 200241 (China)

[3] Prof. X. Guo
State Key Laboratory of Supramolecular Structure and Materials, College of Chemistry and Key Laboratory for Molecular Enzymology and Engineering of the Ministry of Education, College of Life Science
Jilin University
Qianjin Road 2699, Changchun 130012 (China)
E-mail: guoxh@jlu.edu.cn

[4] Prof. Y. Yang
State Key Laboratory of Bioreactor Engineering, Shanghai Key Laboratory of Chemical Biology, School of Pharmacy
East China University of Science and Technology
Meilong Road 130, Shanghai 200237 (China)
Supporting information for this article is given via a link at the end of the document.

Table of Contents

1. Supplementary materials and instrumentation

2. Supplementary experimental methods

3. Supplementary figures and tables

3.1 The construction and characterization of efficient AchE Raman probe (Figure S1).

3.2 ^{1}H NMR, ^{13}C NMR and HR-MS of Compound 2-4, Compound 8, Compound ET-OH, ET-R (R=1-7) (Figure S2-S38).

3.3 The analytical performance of ET-R molecules. (Figure S39-43).

3.4 The recognition mechanism of AuNSs@GSH@ET-R toward AchE (Figure S44-46).

3.5 Stability and biosafety of the develop AuNSs@GSH@ET-5 (Figure S47, 48).

3.6 The ability of AchE to regulate the NSCs differentiation and proliferation (Figure S49).

3.7 SEM images of TFs with different angles (Figure S50)

3.8 The co-localization and biocompatibility of the optical fiber array (Figure S51).

3.9 The Raman spectra of AuNSs@GSH@ET-5 in 24 brain regions of AD mice (Figure S52).

4. Supplementary tables

5. Supplementary references

1. Supplementary materials and instrumentation

1.1 Chemical and Reagents. 3-bromophenylhydrazine hydrochloride, methyl-2-butanone (MIPK), trimethylethynyl silicon, bistriphenylphosphonium palladium dichloride ($PdCl_2(PPh_3)_2$), p-hydroxybenzaldehyde (PHB), potassium carbonate (K_2CO_3), p-methylbenzoyl chloride (pTA), trifluoromethanesulfonic acid (TfOH), 3-(4,5-dimethyl-2-thiazolyl)-2,5-diphenyl-2H-tetrazolium bromide (MTT) were purchased from Aladdin Chemistry Co. Ltd. (China). Dichloromethane (CH_2Cl_2), dimethyl sulfoxide (DMSO), ethyl acetate, acetonitrile, methanol (CH_3OH), sodium hydroxide (NaOH), potassium hydroxide (KOH), CuI, acetic acid (AcOH), triethylamine (NEt_3), methyl iodide (MeI) were obtained from Sinopharm Chemical Reagent Co. Ltd (China). Phosphate buffered saline (PBS), and 0.25% trypsin (with EDTA) were obtained from Hyclone Laboratories Company (U.S.A.). Penicillin (100 μg mL^{-1}) were purchased from Gibco Life Technologies Company (U.S.A.). Amino acid and bio-enzyme were all obtained from Sigma-Aldrich (U.S.A.). All chemicals were analytical-grade and were used without further purification. All samples were prepared with ultrapure water purified by a Milli-Q water gradient system. For the selective testing, Hydroxyl radical (•OH) was produced by the Fenton reaction of Fe^{2+} (10 μM) with H_2O_2 (60 μM). $^1O_2$ was produced from the reaction of H_2O_2 (10 μM) with NaClO (10 μM). Peroxynitrite ($ONOO^-$) was generated by the reaction between $NaNO_2$ (10 μM) and H_2O_2 (10 μM). (ROO•) was derived from thermolysis of AAPH (10 μM) dissolved in air-saturated aqueous solution at 37 ºC.

1.2 Instruments. The nuclear magnetic resonance (NMR) spectrum was collected on a Bruker 500 MHz spectrometer (Bruker, Germany). Mass spectrum (MS) was obtained on an Agilent 6890 spectrometer (Agilent, USA). UV−vis absorption spectrum was recorded on a UH5300 spectrophotometer (Hitachi, Japan). The TEM image of AuNSs was characterized with a JEM-2100F transmission electron microscope (JEOL, Japan). Fourier transform infrared spectroscopy (FTIR) spectra was collected on a Nicolet iS10 FTIR spectrometer (Thermo Fisher scientific, USA). Cell apoptosis assay experiment and fluorescence-activated cell sorting for NSCs were conducted by using an Image Stream

mkII system (Merk Serono Co., Ltd., Germany). Fluorescence confocal imaging was measured by Leica TCS-SP8 confocal laser scanning microscope (Leica, Germany).

2. Supplementary experimental methods

2.1 Synthesis of AchE recognition molecule: 5-ethynyl-1,2,3,3-tetramethyl-based molecules (ET-R, R=1-7).

Compound 1: 6.7mmol of 3-bromophenylhydrazine hydrochloride and 13.5 mmol of 3-methyl-2-butanone (MIPK) were added to a three-necked flask containing 12 mL of acetic acid (AcOH), and heated to 120 °C in nitrogen atmosphere for 7 h. The reaction was then stirred at room temperature for 6 h. After the reaction, the solution was evaporated, and then ether and water were added for liquid separation treatment. The organic phase was washed with 10 % KOH aqueous solution and distilled water. Then, the solution was dried with anhydrous sodium sulfate (Na_2SO_4), and the black brown oily product was obtained by evaporation.

Compound 2: 4.2 mmol of compound 1 and 8.4 mmol of trimethylethynyl silicon were added to 12 mL of triethylamine (NEt_3), and then 0.0884 g of bistriphenylphosphonium palladium dichloride ($PdCl_2(PPh_3)_2$) and 0.024 g of cuprous iodide (CuI) were added to the mixed solution, and refluxed at 100 ºC for 2.5 h under nitrogen atmosphere. After the reaction, the solution was extracted with ethyl acetate (EA), and evaporated to obtain a mixture. Purify by column chromatography with petroleum ether (PE): ethyl acetate (EA)=10:1 to obtain compound 2.

Compound 3: 0.786 g compound 2 was added in the flask, then add 436 μL methyl iodide (MeI) and 6 mL acetonitrile (MeCN), refluxed at 90 °C for 5 hours. After the reaction, the solution was directly dried, and then the compound 3 was purified by column chromatography with PE: EA = 20 : 1.

Compound 4: 198.7 mg of compound 3 and 73.3 mg of p-hydroxybenzaldehyde (PHB) were dissolved in 5 mL of ethanol (EtOH), heated to 85 °C in nitrogen atmosphere and refluxed for 5.5 h. After the reaction, the solution was directly dried, and then the molecule compound 4 was purified by column chromatography with dichloromethane (CH_2Cl_2) : methanol (MeOH) = 10 : 1.

Compound ET-OH: 2 g of compound 4 and 5.5 g potassium carbonate (K_2CO_3) dissolved in 40 mL anhydrous methanol, then added 40 μL dichloromethane (CH_2Cl_2), stirring overnight at room temperature. The reaction mixture was dried directly, and then the compound ETH was obtained by column chromatography with CH_2Cl_2 : MeOH = 10 : 1.

ET-R (R=1, 2, 3, 5) molecules: 1eq. compound ET-OH, 6 eq. formyl chloride (1) / Dimethylcarbamoyl chloride (2) / p-methylbenzoyl chloride (3) / 4-(Dimethylamino) benzoyl chloride (5), and 1% trifluoromethanesulfonic acid (TfOH) were added into anhydrous acetonitrile (CH_3CN), stirring overnight at room temperature. The mixture was dried, and then purified by column chromatography with CH_2Cl_2 : MeOH = 10 : 1 to obtain ET-R (R=1, 2, 3, 5).

ET-4 and ET-6 molecules: 1 eq. of p-dimethylaminobenzoic acid and 1 eq. of 3-hydroxypropionaldehyde (4) / 4-Hydroxycinnamaldehyde (6) were added to a dichloromethane and tetrahydrofuran mixed solvent (1:1), and then stirred at room temperature for 1 h. Dicyclohexylcarbodiimide (DCC, 0.12 eq.) and 4-dimethylaminopyridine (0.072g) were added to the mixture and the reaction was stirred at room temperature for 10 h. The reaction mixture was dried directly, and then washed with 10% HCl and 5% KOH for three times, respectively. Then the white compound was obtained by rotary evaporation. White compound (1g) and compound 3 (500 mg) were added to 5 ml of acetic anhydride and then heated to 60 ℃ for 10 hours. Furthermore, 50 ml of petroleum ether was added and then reddish-brown solid powder was precipitated. The red-brown powder was washed three times with petroleum ether and ethyl acetate and dried. 50mg red-brown powder dissolved in 5ml of ethanol, add 50uL of tetrabutylammonium fluoride and stir at room temperature for 48 hours. The mixture was dried, and then purified by column chromatography with CH_2Cl_2 : MeOH = 1 : 1 to obtain ET-4 and ET-6, respectively.

ET-7 molecule: ET-5 molecule (0.01 mmol) was dissolved in ethanol (1 mL), and then NaBH4 (0.04 mmol in 0.5 mL ethanol) was added drop-wise over 10 min. Subsequently, the reaction mixture was stirred at room temperature for 20 min, and the solvent was removed under reduced pressure. The resulting residue was purified on a silica gel column

(CH_2Cl_2 /petroleum ether = 1: 5) to afford ET-7 molecule.

2.2 Synthesis of gold stars (AuNSs). Sodium citrate solution (15 mL 1 %) was added to 100 mL boiled 1 mM chloroauric acid solution, and stirred until the solution became wine red. The solution was cooled to room temperature to prepare gold seed solution, which was stored in a refrigerator at 4 °C for further using. Gold seeds (500 μL) were mixed with 50 mL 0.25 mM chloroauric acid solution, and then 50 μL 1M HCl solution was added to adjust pH to about 3. Then, 500 μL silver nitrate solution (1 mM) and 250 μL 0.1 M ascorbic acid solution were added to the above solution and stirred for 30 s. Then, 20 mL 3 mM sodium citrate solution was added to the above solution. After rapid mixing, it was centrifuged at 4500 rpm for 15 min. Followed by the supernatant was removed, the particles were dispersed in 3 mL 3 mM sodium citrate and stored in a refrigerator at 4 °C for further use.

2.3 Synthesis of AuNSs@GSH@ET-R. Glutathione solution (2 μL 10 mM) was added into 200 μL prepared AuNSs solution, and the reaction was oscillated overnight at room temperature. Then, the solution was centrifuged at 4500 rpm for 15 min to remove the supernatant, and the particles were re-dispersed in 200 μL 3 mM sodium citrate to synthesize AuNSs@GSH probe. After that, 2 μL 1 mM ET-R ethanol solution was added to the prepared 200 μL AuNSs@GSH solution. After deoxygenation for 30 min, the mixed solution was reacted at 60 °C for 5 h. Next, the solution was centrifuged at 4500 rpm for 15 min, the supernatant was removed, and then the particles were re-dispersed in 200 μL sodium citrate (3 mM) to synthesize the AuNSs@GSH@ET-R probe.

2.4 Neural Stem Cell (NSCs) culture. Neural stem cell (NSC) isolation and culture according the literature [1]. The embryos of pregnant C57/BL-6 wild mice were dissected and put into precooling HBSS buffer. The brain was dissected under the microscope. After stripping the meningeal and blood vessels, the tissue was cut into small pieces and filtered. The filtered cells were collected and dispersed in the proliferation medium and cultured in 37 °C under 5% CO_2 before using.

2.5 Morris water maze. Morris water maze (MWM) is mainly used to test the learning and memory ability of experimental animals [2]. Briefly, AD mice (20-weeks) were placed in water toward the wall of the pond. Record the time when animals found the underwater platform. In the early stages of training, if this time is more than 150 s, the animals will be guided to the platform, allowing animals to stay on the platform for 10 s. Each animal was trained five times a day, and the interval between the two trainings was 20-30 min for six consecutive days. The time of animals reaching the platform was recorded on the seventh day as the detection index of spatial memory.

2.6 Ray-tracing simulation. We used commercial optical ray-tracing software Comsol to design and simulate the performances of TFs. The single TF was modeled as a straight core/cladding segment followed by a conical taper. The materials forming all the components of the TFs and the surrounding media were assumed to be homogenous (refractive index constant in space). The core/cladding diameters were 100/30 μm for fibers with numerical apertures NA = 0.22, NA = 0.37 and NA=0.48, respectively (Aunion).

3. Supplementary figures and tables

3.1 The construction and characterization of efficient AchE Raman probe.

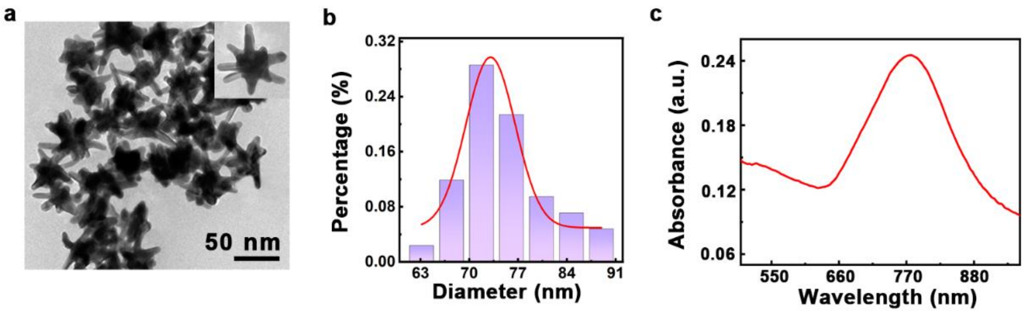

Figure S1. (a) TEM images of AuNSs. Inset: a single nanoparticle. (b) The diameter distribution of AuNSs. (c) UV-vis spectrum of AuNSs (15 μg mL^{-1}).

3.2 Synthesis diagram of ET-5.

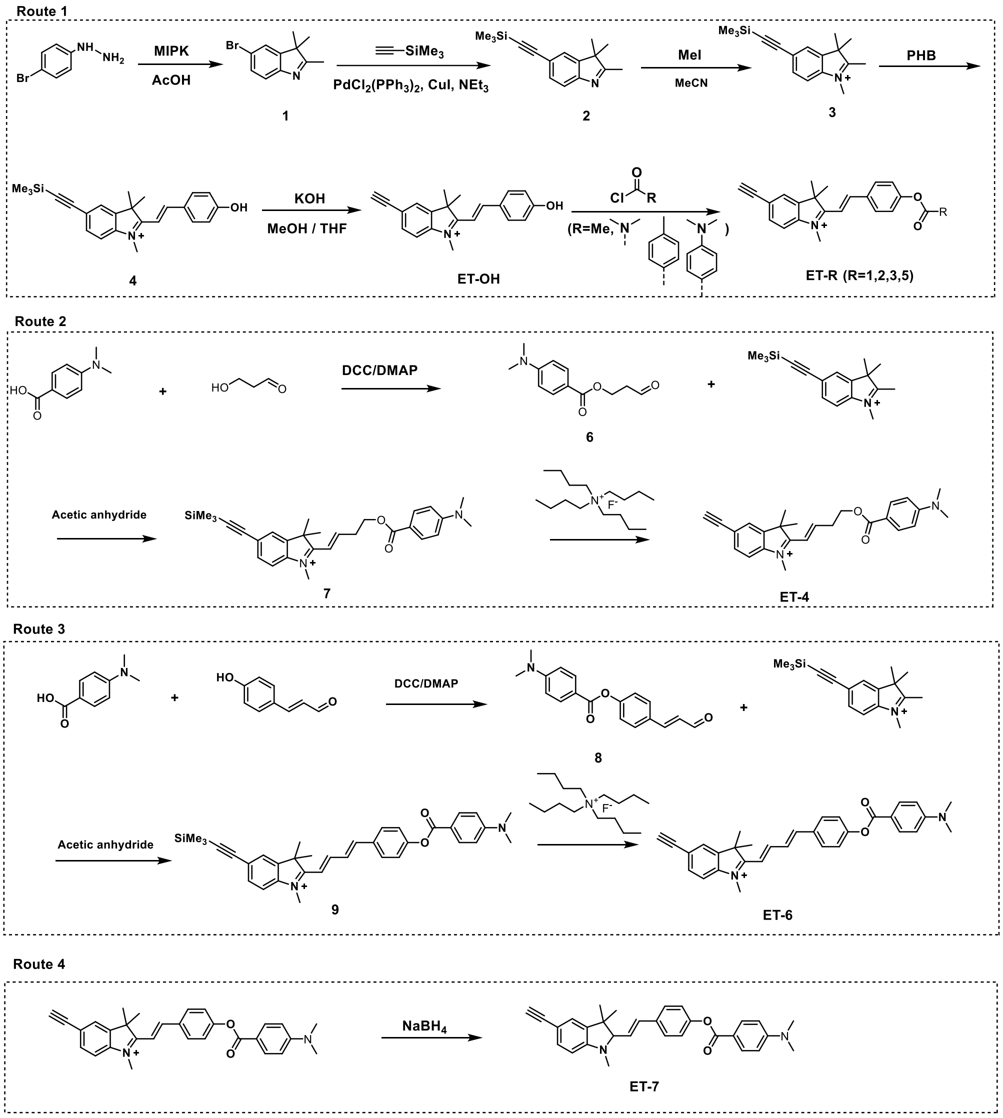

Figure S2. Synthesis diagram of ET-R.

3.2. ^{1}H NMR, ^{13}C NMR and HR-MS of Compound 2-4, Compound 8, Compound ET-OH, ET-R (R=1-7).

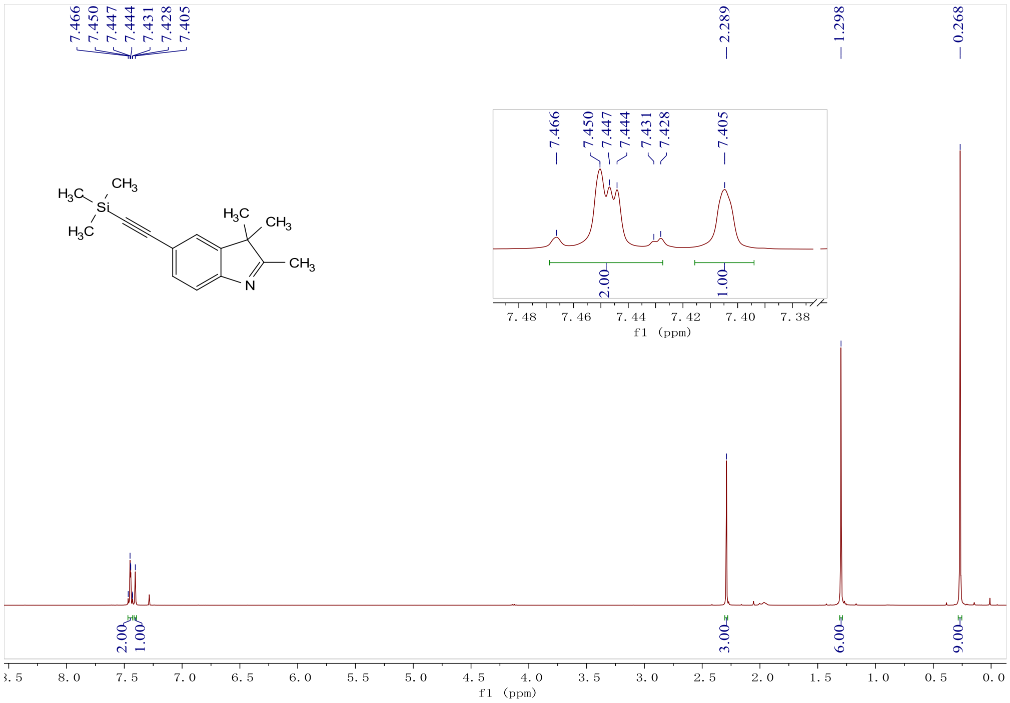

Figure S3. ^{1}H NMR spectrum (500 MHz) of compound 2 in $CHCl_3$-d_1. δ: 7.466-7.405 (m, 2H), 7.405 (s, 1H), 2.289 (s, 3H), 1.298 (s, 6H), 0.268 (s, 9H).

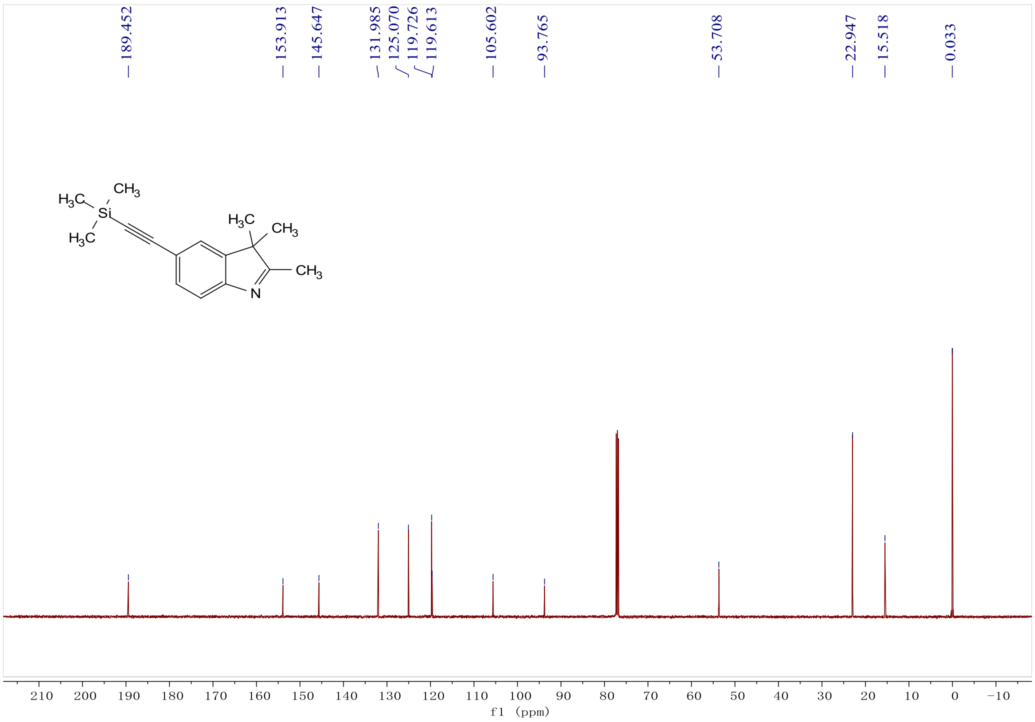

Figure S4. ^{13}C NMR spectrum (125 MHz) of compound 2 in $CHCl_3$-d_1. δ= 189.45, 153.91, 145.65, 131.99, 125.07, 119.73, 119.61, 105.60, 93.76, 53.71, 22.95, 15.52, 0.03 ppm.

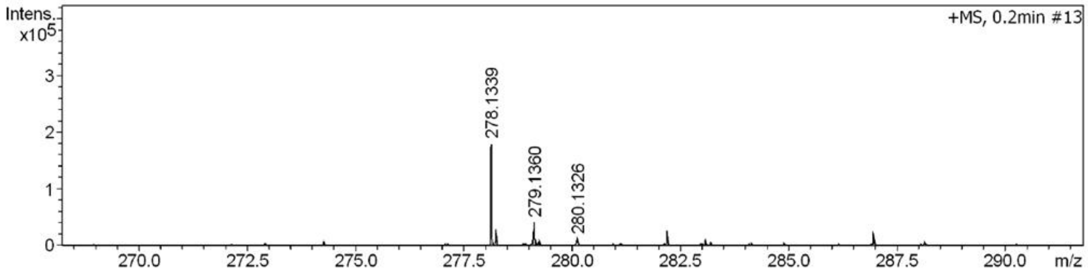

#	m/z	Res.	S/N	I	I %	FWHM
1	278.1339	17129	352.5	178760	100.0	0.0162
2	279.1360	14977	80.3	40872	22.9	0.0186
3	280.1326	9136	20.7	10600	5.9	0.0307

Meas. m/z	#	Ion Formula	m/z	err [ppm]	mSigma	#	Score	rdb	e⁻ Conf	N-Rule
278.1339	1	C16H21NNaSi	278.1335	-1.2	3.4	1	100.00	7.5	even	ok

Figure S5. MS spectrum of compound 2.

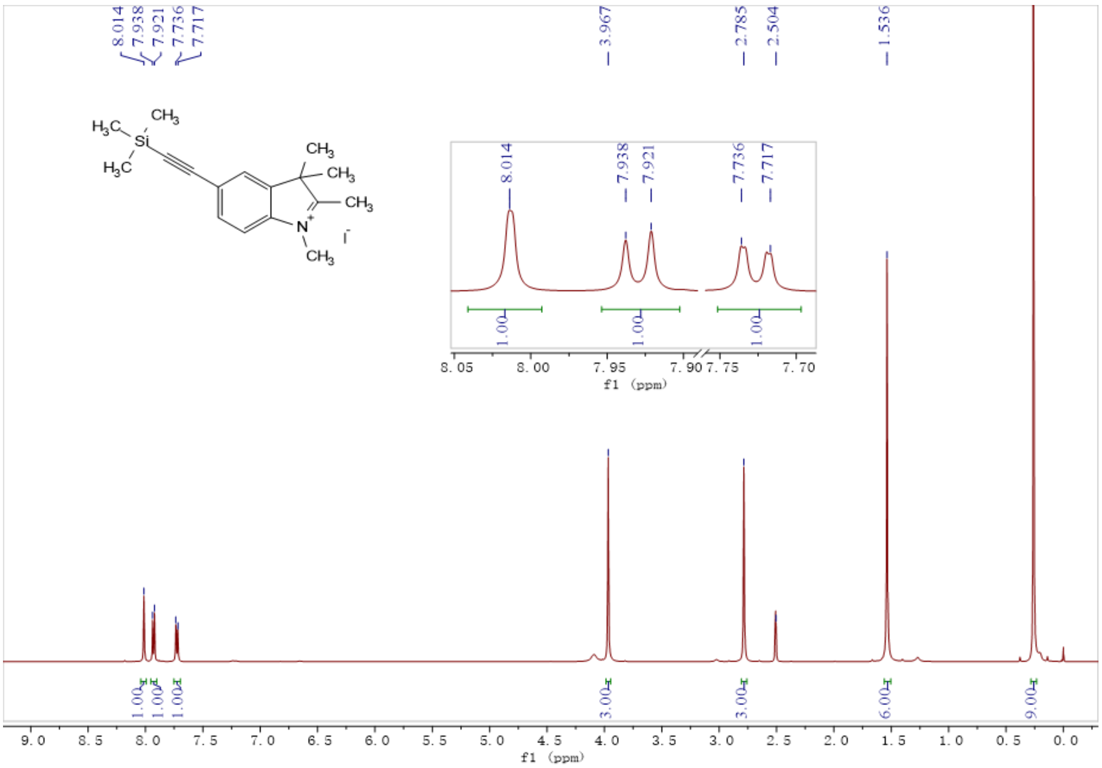

Figure S6. ^{1}H NMR spectrum (500 MHz) of compound 3 in DMSO-d_6. δ: 8.014 (s, 1H), 9.938-7.921 (m, 1H), 7.736-7.717 (m, 1H), 3.967 (s, 3H), 2.785 (s, 3H), 1.536 (s, 6H), 0.259 (s, 9H).

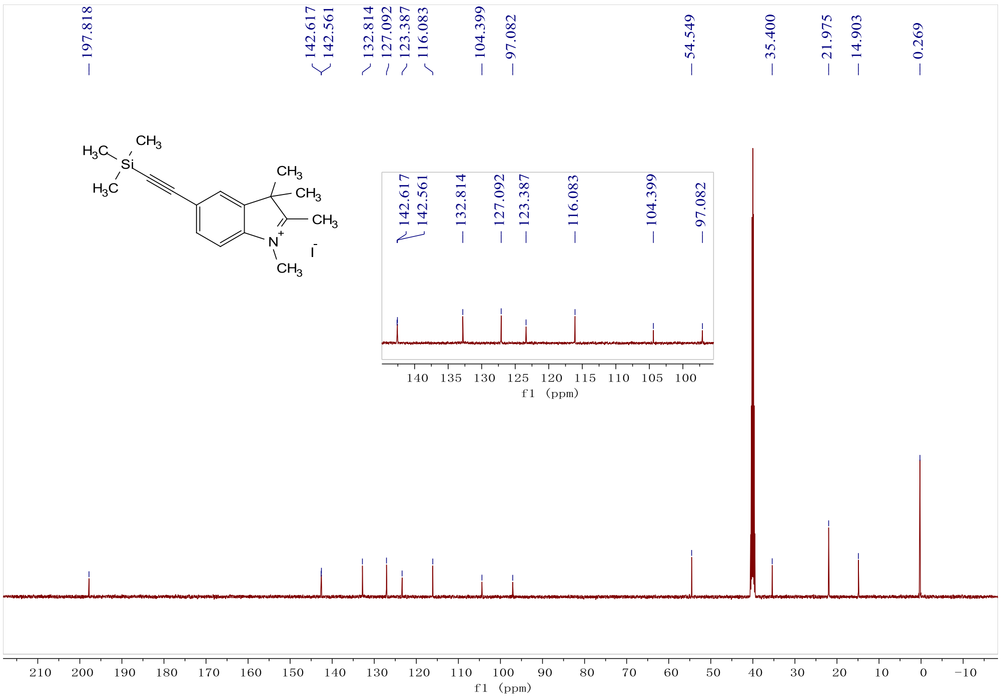

Figure S7. ^{13}C NMR spectrum (125 MHz) of compound 3 in DMSO-d_6. δ= 197.82, 142.62, 142.56, 132.81, 127.09, 123.39, 116.08, 104.40, 97.08, 54.55, 40.18, 35.40, 21.98, 14.90, 0.27 ppm.

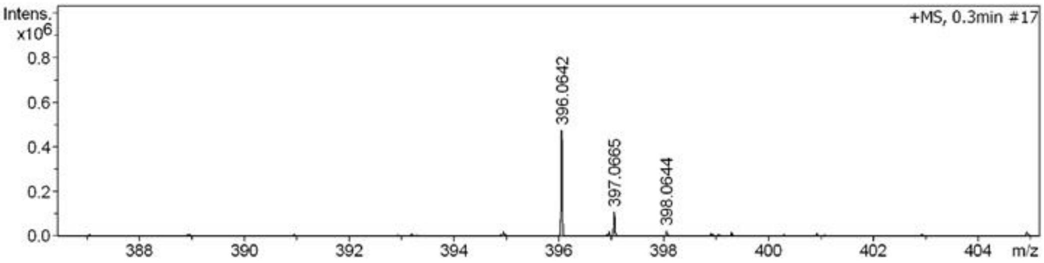

#	m/z	Res.	S/N	I	I %	FWHM
1	396.0642	22857	818.4	473352	100.0	0.0173
2	397.0665	19390	185.4	107348	22.7	0.0205
3	398.0644	13246	37.4	21644	4.6	0.0301

Meas. m/z	#	Ion Formula	m/z	err [ppm]	mSigma	#	Score	rdb	e⁻ Conf	N-Rule
396.0642	1	C17H23INSi	396.0639	-0.8	10.2	1	100.00	7.5	even	ok

Figure S8. MS spectrum of compound 3.

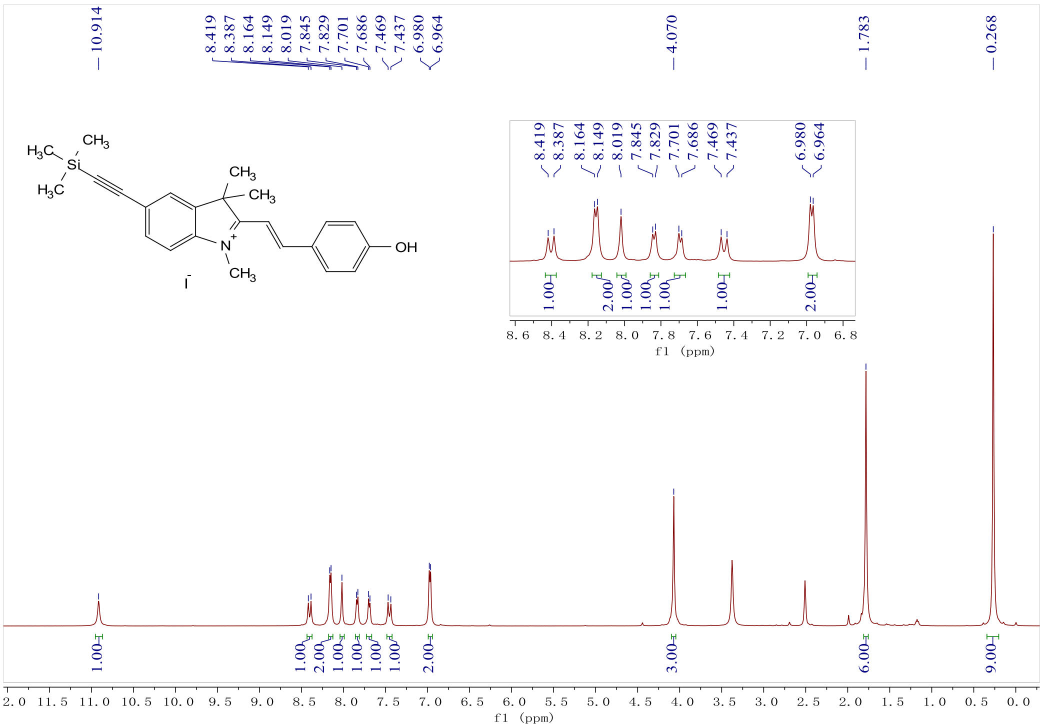

Figure S9. ^{1}H NMR spectrum (500 MHz) of compound 4 in DMSO-d_6. δ 10.914 (s, 1H), 8.403 (d, J = 16.0 Hz, 1H), 8.156 (d, J = 7.6 Hz, 2H), 8.019 (s, 1H), 7.837 (d, J = 7.8 Hz, 1H), 7.694 (d, J = 7.9 Hz, 1H), 7.453 (d, J = 16.0 Hz, 1H), 6.972 (d, J = 7.7 Hz, 2H), 4.070 (s, 3H), 1.783 (s, 6H), 0.268 (s, 9H).

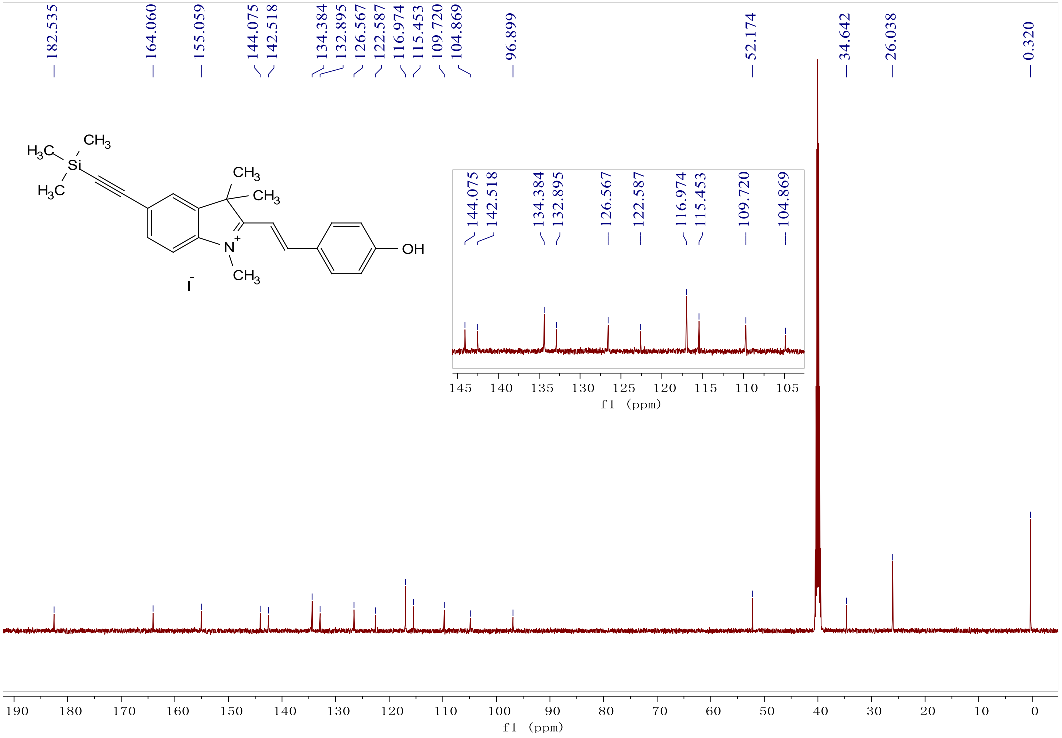

Figure S10. ^{13}C NMR spectrum (125 MHz) of compound 4 in DMSO-d_6. δ= 182.53, 164.06, 155.06, 144.08, 142.52, 134.38, 132.89, 126.57, 122.59, 116.97, 115.45, 109.72, 104.87, 96.90, 52.17, 34.64, 26.04, 0.32 ppm.

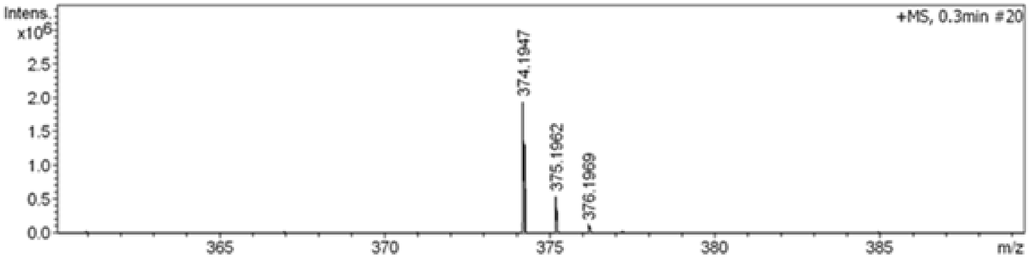

#	m/z	Res.	S/N	I	I %	FWHM
1	374.1947	15625	2625.7	1941572	100.0	0.0239
2	375.1962	22643	741.4	548544	28.3	0.0166
3	376.1969	9252	148.8	110160	5.7	0.0407

Meas. m/z	#	Ion Formula	m/z	err [ppm]	mSigma		Score	rdb	e$^-$ Conf	N-Rule
374.1947	1	C24H28NOSi	374.1935	-3.3	22.1	1	100.00	12.5	even	ok

Figure S11. MS spectrum of compound 4.

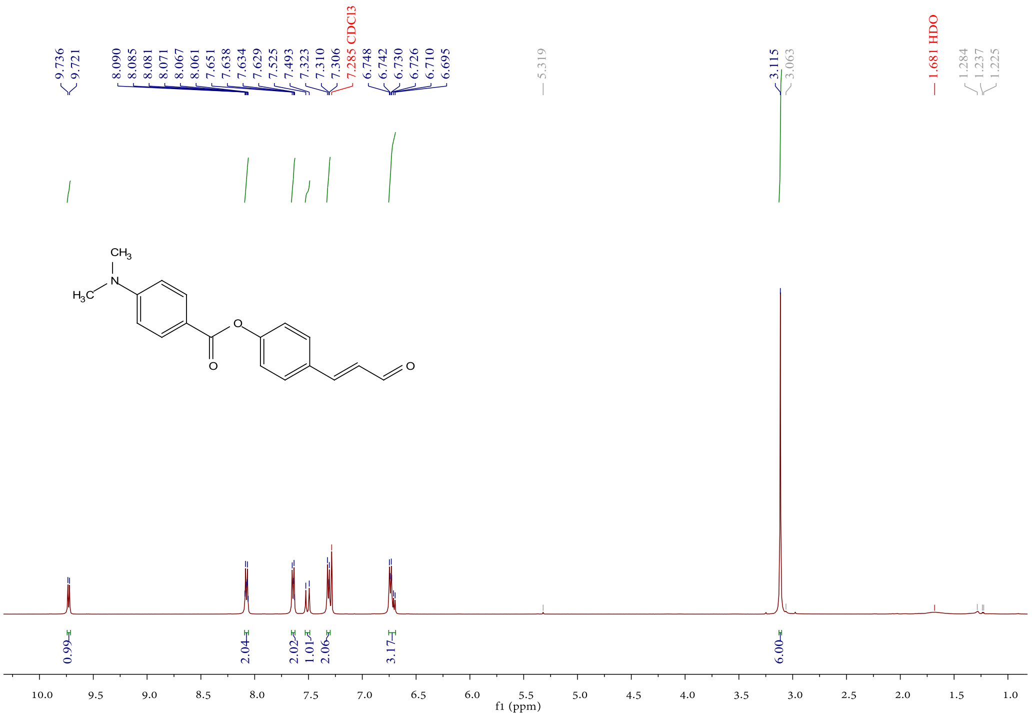

Figure S12. ^{1}H NMR spectrum (500 MHz) of compound 8 in $CHCl_3$-d_1. δ 9.728 (d, *J* = 7.7 Hz, 1H), 8.094 - 8.058 (m, 2H), 7.659 - 7.626 (m, 2H), 7.509 (d, *J* = 16.0 Hz, 1H), 7.315 (d, *J* = 8.5 Hz, 2H), 6.755 - 6.690 (m, 3H), 3.115 (s, 6H).

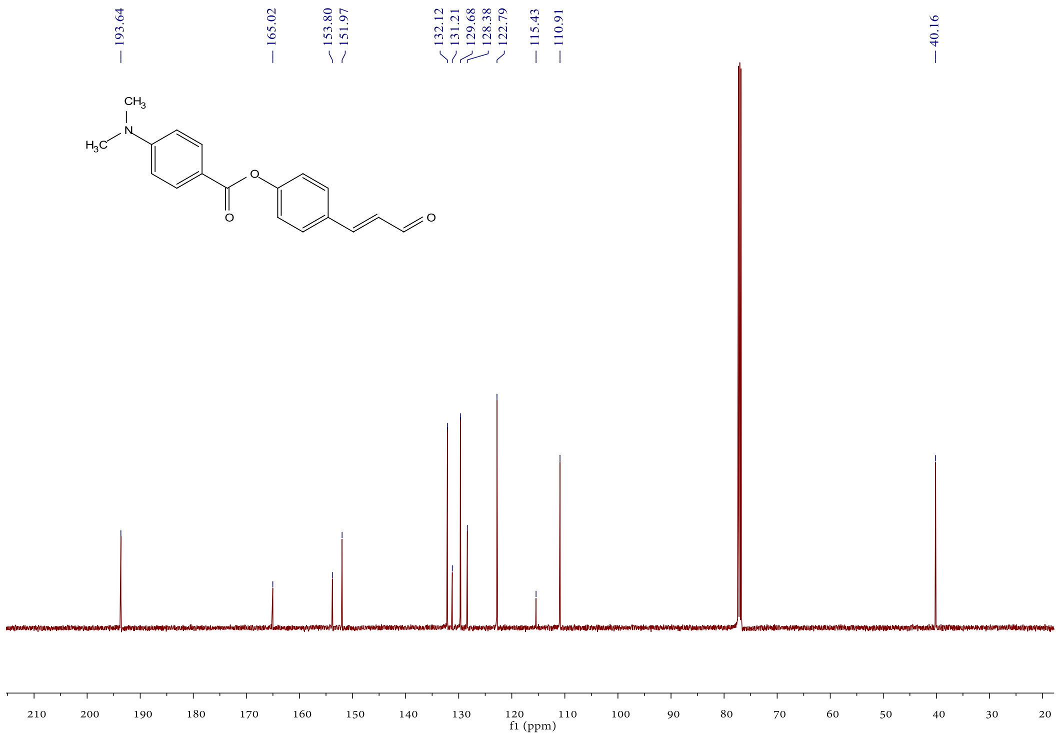

Figure S13. ^{13}C NMR spectrum (125 MHz) of compound 8 in $CHCl_3$-d_1.δ 193.64, 165.02, 153.80, 151.97, 132.12, 131.21, 129.68, 128.38, 122.79, 115.43, 110.91, 40.16.

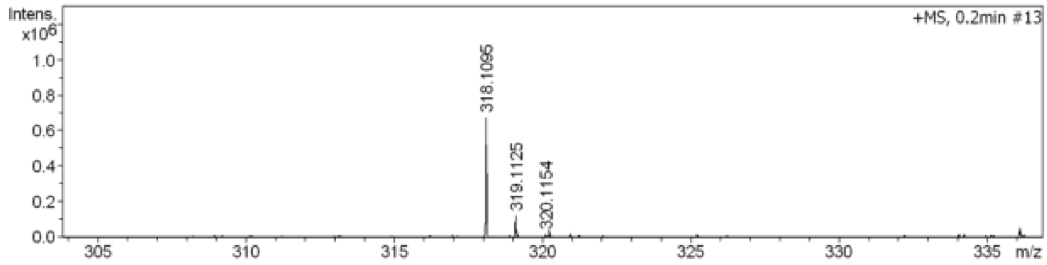

#	m/z	Res.	S/N	I	I %	FWHM
1	318.1095	20450	536.5	671884	100.0	0.0156
2	319.1125	14593	94.5	118388	17.6	0.0219
3	320.1154	13309	11.7	14712	2.2	0.0241

Meas. m/z	#	Ion Formula	m/z	err [ppm]	mSigma		Score	rdb	e⁻ Conf	N-Rule
318.1095	1	C18H17NNaO3	318.1101	1.9	14.7	1	100.00	10.5	even	ok

Figure S14. MS spectrum of compound 8.

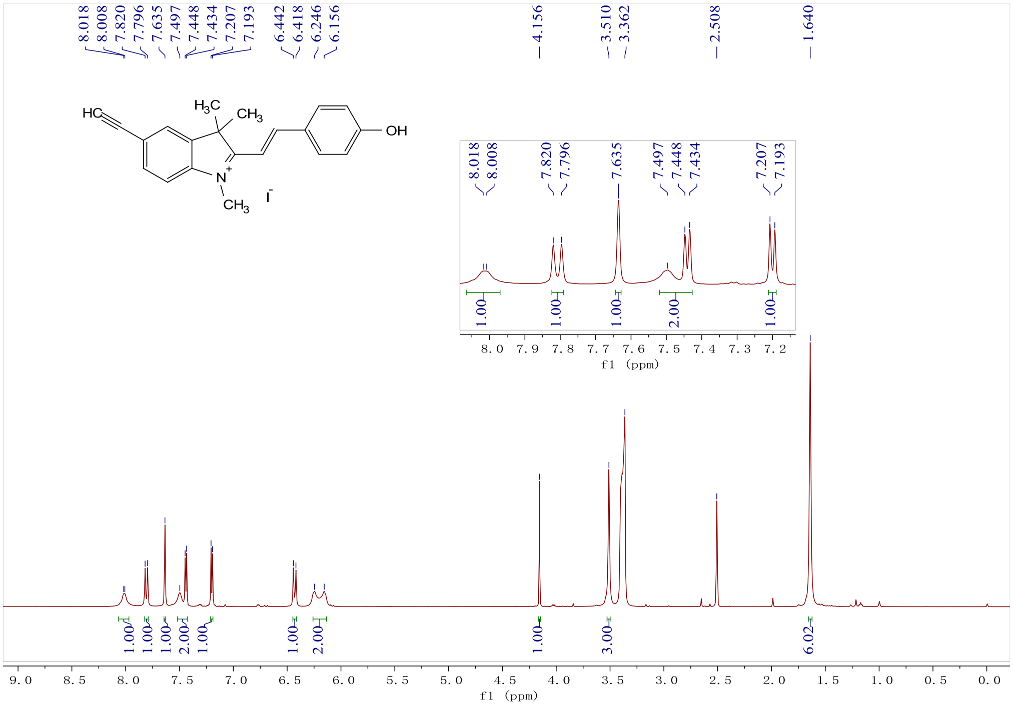

Figure S15. ^{1}H NMR spectrum (500 MHz) of compound ET-5OH in DMSO-d_6. δ 8.008-8.018 (m, 1H), 7.808 (d, J = 12.0 Hz, 1H), 7.635 (s, 1H), 7.497-7.505 (m, 1H), 7.441 (d, J = 7.0 Hz, 1H), 7.200 (d, J = 7.0 Hz, 1H), 6.430 (d, J = 12.0 Hz, 1H), 6.156-6.246 (m, 2H), 4.156 (s, 1H), 3.510 (s, 3H), 1.640 (s, 6H).

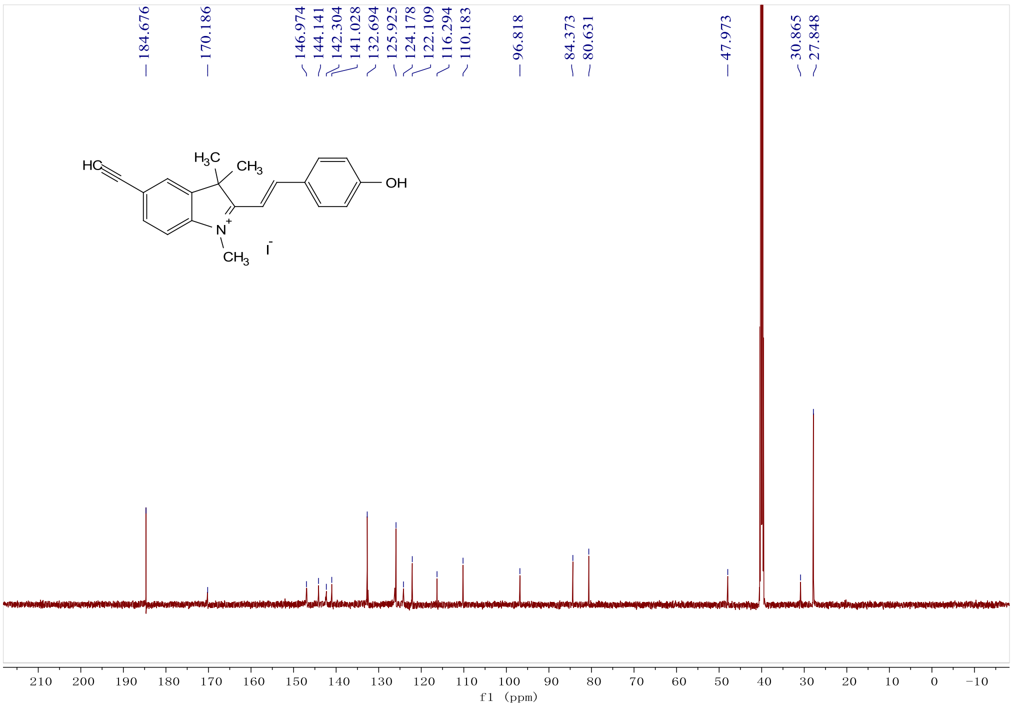

Figure S16. ^{13}C NMR spectrum (125 MHz) of compound ET-5OH in DMSO-d_6. δ 184.68, 170.19, 146.97, 144.14, 142.30, 141.03, 132.69, 125.92, 124.18, 122.11, 116.29, 110.18, 96.82, 84.37, 80.63, 47.97, 30.86, 27.85 ppm.

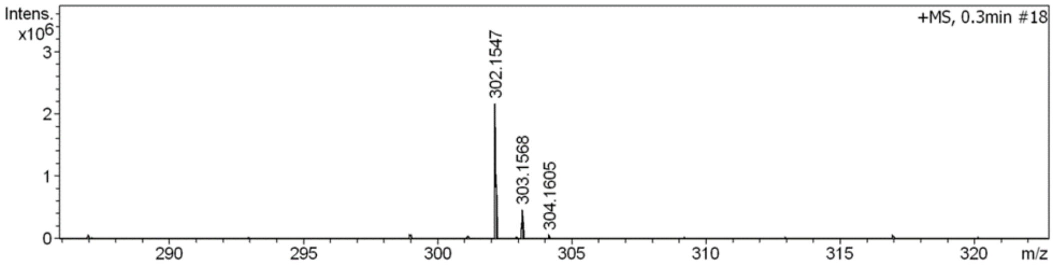

#	m/z	Res.	S/N	I	I %	FWHM
1	302.1547	14210	3170.6	2153852	100.0	0.0213
2	303.1568	20012	684.6	465972	21.6	0.0151
3	304.1605	12588	68.3	46540	2.2	0.0242

Meas. m/z	#	Ion Formula	m/z	err [ppm]	mSigma	#	Score	rdb	e⁻ Conf	N-Rule
302.1547	1	C21H20NO	302.1539	-2.4	10.6	1	100.00	12.5	even	ok

Figure S17. MS spectrum of ET-5OH.

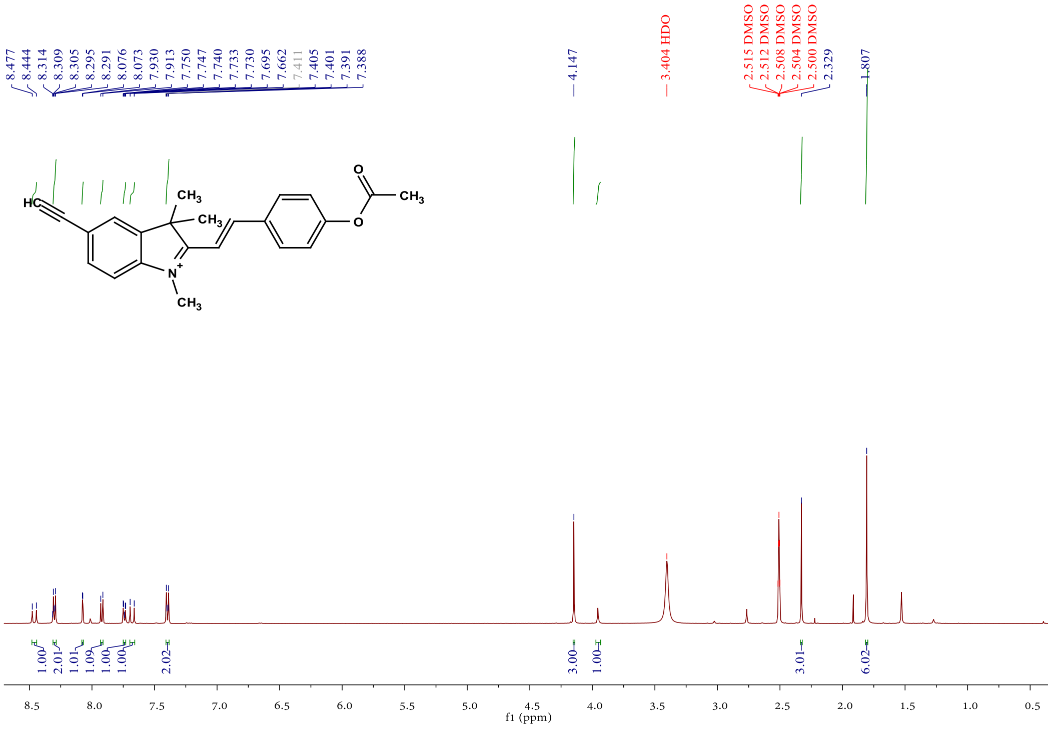

Figure S18. ^{1}H NMR spectrum (500 MHz) of compound ET-1 in DMSO-d_6. δ 8.461 (d, J = 16.4 Hz, 1H), 8.313 – 8.282 (m, 2H), 8.075 (d, J = 1.5 Hz, 1H), 7.921 (d, J = 8.4 Hz, 1H), 7.757 – 7.723 (m, 1H), 7.679 (d, J = 16.4 Hz, 1H), 7.416 – 7.374 (m, 2H), 4.147 (s, 3H), 3.956 (s, 1H), 2.329 (s, 3H), 1.807 (s, 6H).

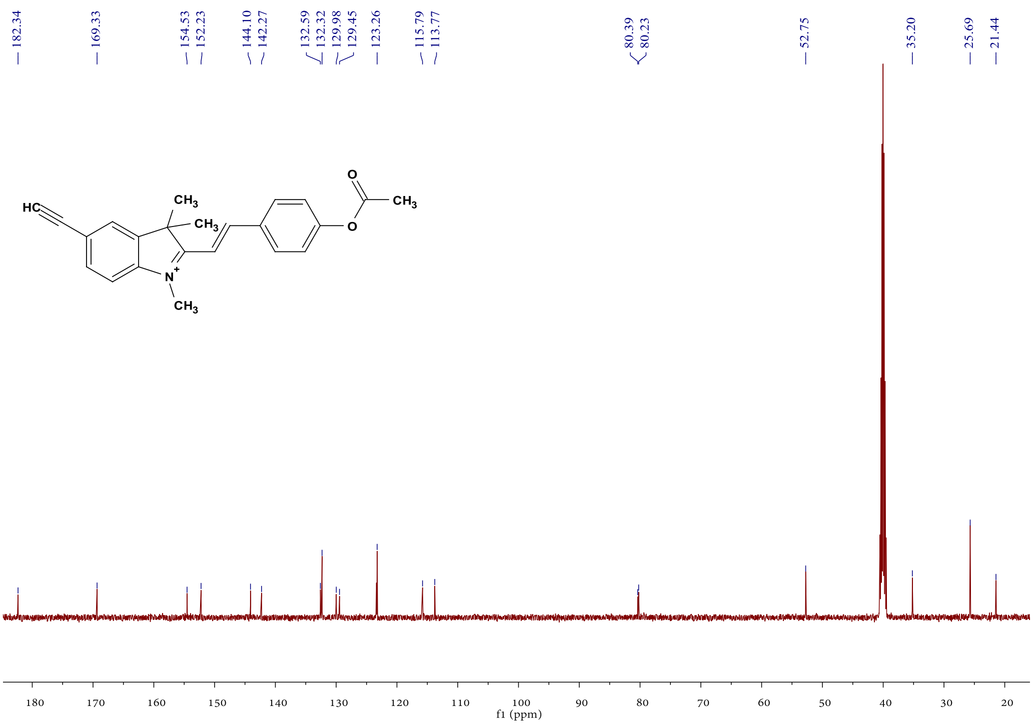

Figure S19. ^{13}C NMR spectrum (125 MHz) of compound ET-1 in DMSO-d_6. δ 182.34, 169.33, 154.53, 152.23, 144.10, 142.27, 132.59, 132.32, 129.98, 129.45, 123.26, 115.79, 113.77, 80.39, 80.23, 52.75, 35.20, 25.69, 21.44.

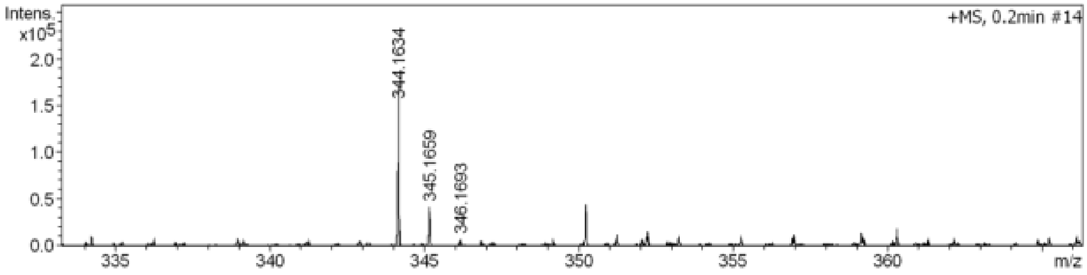

#	m/z	Res.	S/N	I	I %	FWHM
1	344.1634	13629	158.0	150900	100.0	0.0253
2	345.1659	12457	43.9	41936	27.8	0.0277
3	346.1693	13262	6.9	6540	4.3	0.0261

Meas. m/z	#	Ion Formula	m/z	err [ppm]	mSigma		Score	rdb	e⁻ Conf	N-Rule
344.1634	1	C23H22NO2	344.1645	3.3	13.6	1	100.00	13.5	even	ok

Figure S20. MS spectrum of ET-1.

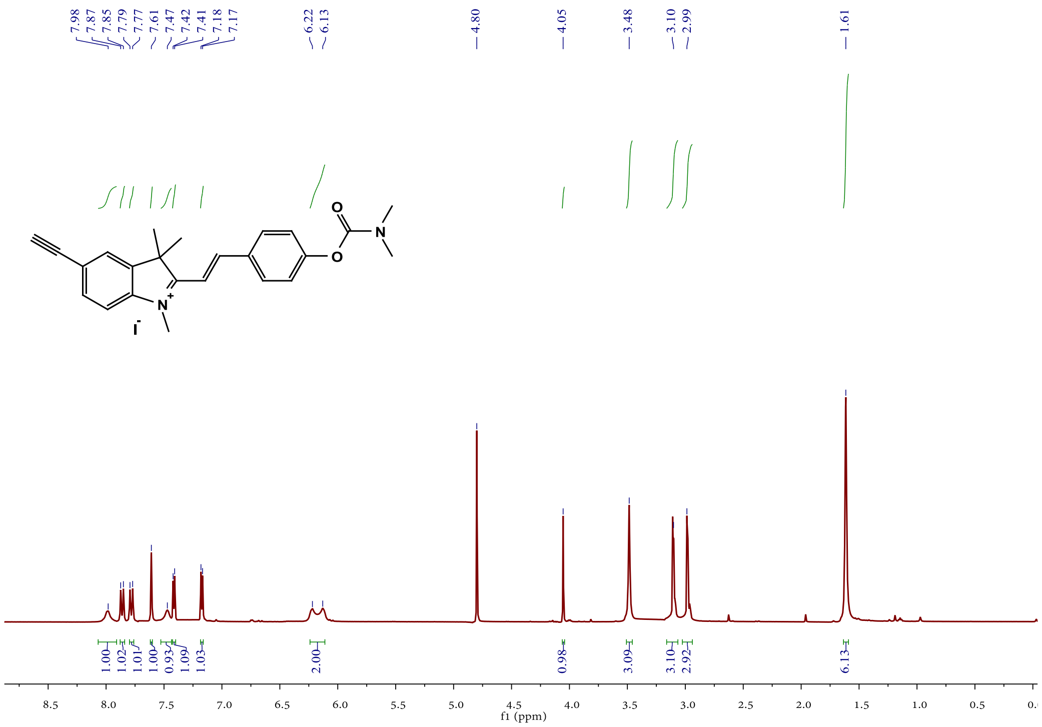

Figure S21. ^{1}H NMR spectrum (500 MHz) of compound ET-2 in CH_3OH-d_4. δ 7.982 (s, 1H), 7.862 (d, J = 9.3 Hz, 1H), 7.782 (d, J = 9.3 Hz, 1H), 7.609 (s, 1H), 7.471 (s, 1H), 7.415 (d, J = 5.4 Hz, 1H), 7.174 (d, J = 5.4 Hz, 1H), 6.174 (d, J = 35.6 Hz, 2H), 4.055 (s, 1H), 3.484 (s, 3H), 3.101 (s, 3H), 2.986 (s, 3H), 1.614 (s, 6H).

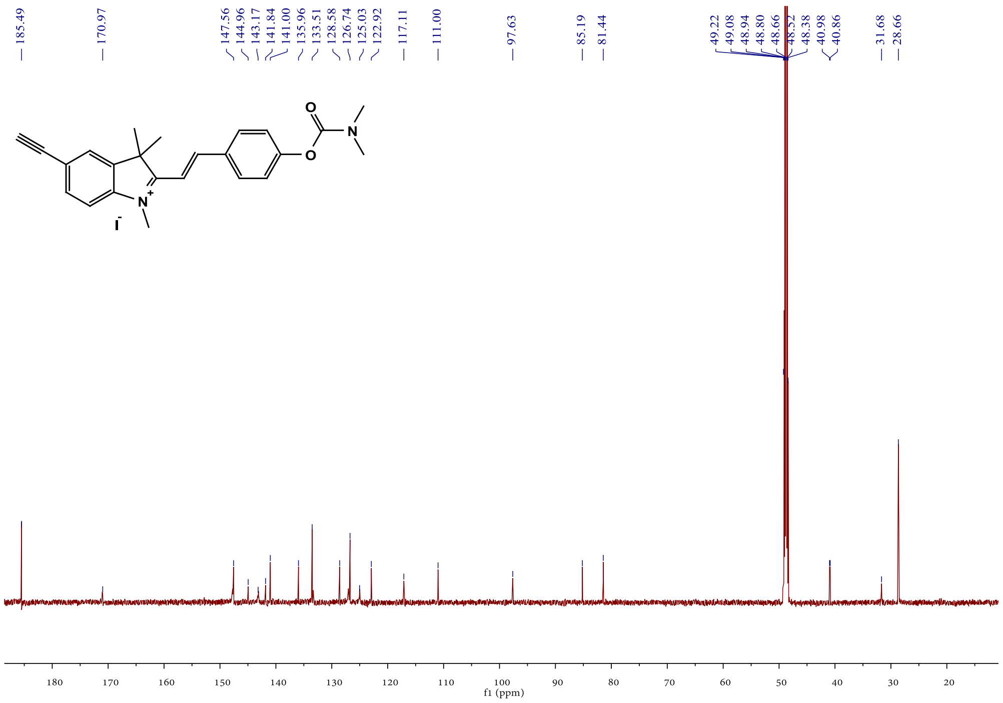

Figure S22. ^{13}C NMR spectrum (125 MHz) of compound ET-2 in CH_3OH-d_4. δ 185.49, 170.97, 147.56, 144.96, 143.17, 141.84, 141.00, 135.96, 133.51, 128.58, 126.74, 125.03, 122.92, 117.11, 111.00, 97.63, 85.19, 81.44, 49.22, 49.08, 48.94, 48.80, 48.66, 48.52, 48.38, 40.98, 40.86, 31.68, 28.66.

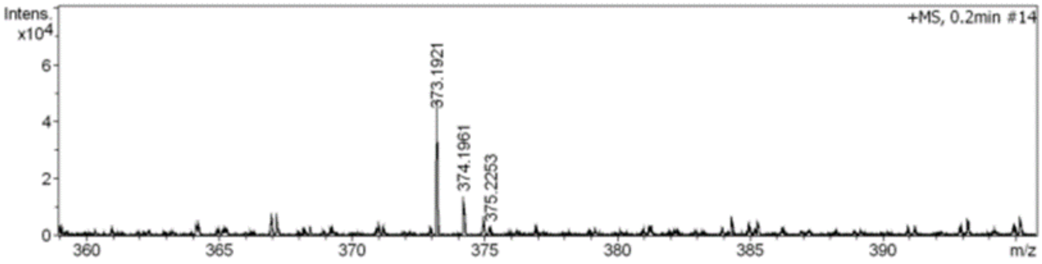

#	m/z	Res.	S/N	I	I %	FWHM
1	373.1921	12271	64.4	42644	100.0	0.0304
2	374.1961	12067	20.9	13816	32.4	0.0310
3	375.2253	14488	4.6	3036	7.1	0.0259

Meas. m/z	#	Ion Formula	m/z	err [ppm]	mSigma	#	Score	rdb	e⁻ Conf	N-Rule
373.1921	1	C24H25N2O2	373.1911	-2.8	33.2	1	100.00	13.5	even	ok

Figure S23. MS spectrum of ET-2.

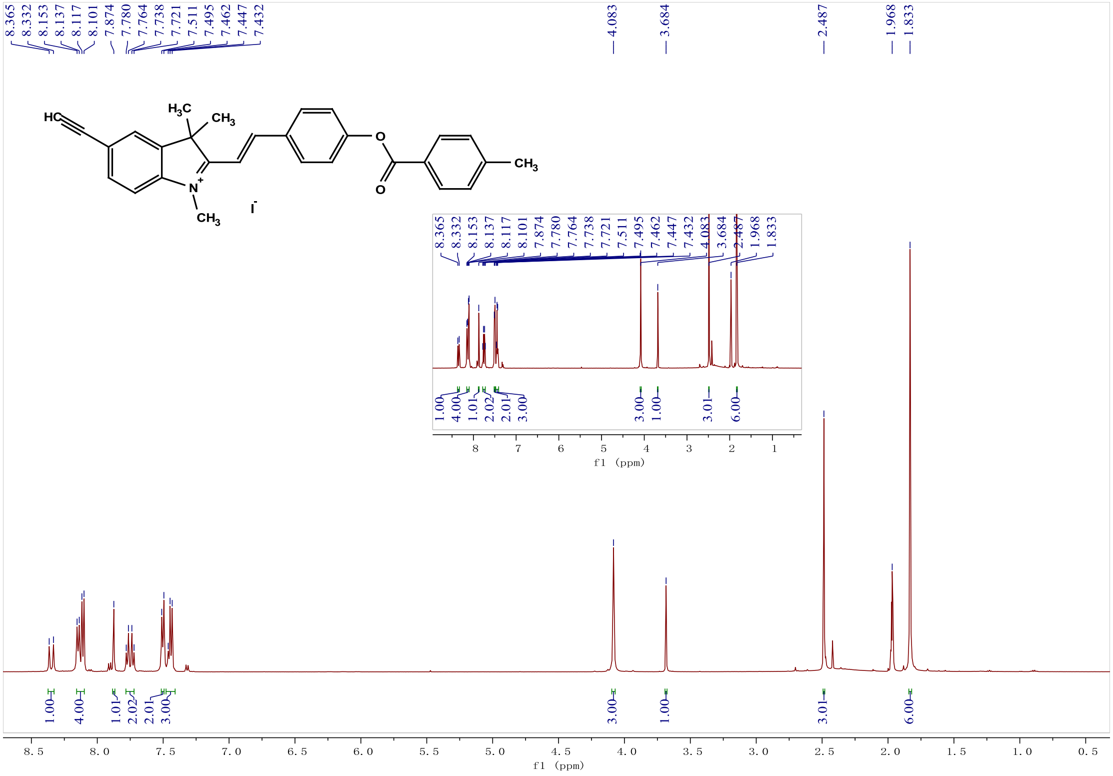

Figure S24. ^{1}H NMR spectrum (500 MHz) of ET-3 in CH_3CN-d_3. δ 8.349 (d, J = 16.5 Hz, 1H), 8.145 (d, J = 8.0 Hz, 1H), 8.109 (d, J = 8.0 Hz, 1H), 7.751 (dd, J = 8.0 Hz, J = 21.5 Hz,2H), 7.503 (d, J = 8.0 Hz, 1H), 7.432-7.462 (m, 3H), 4.083 (s, 3H), 3.684 (s, 1H), 2.487 (s, 3H), 1.833 (s, 6H).

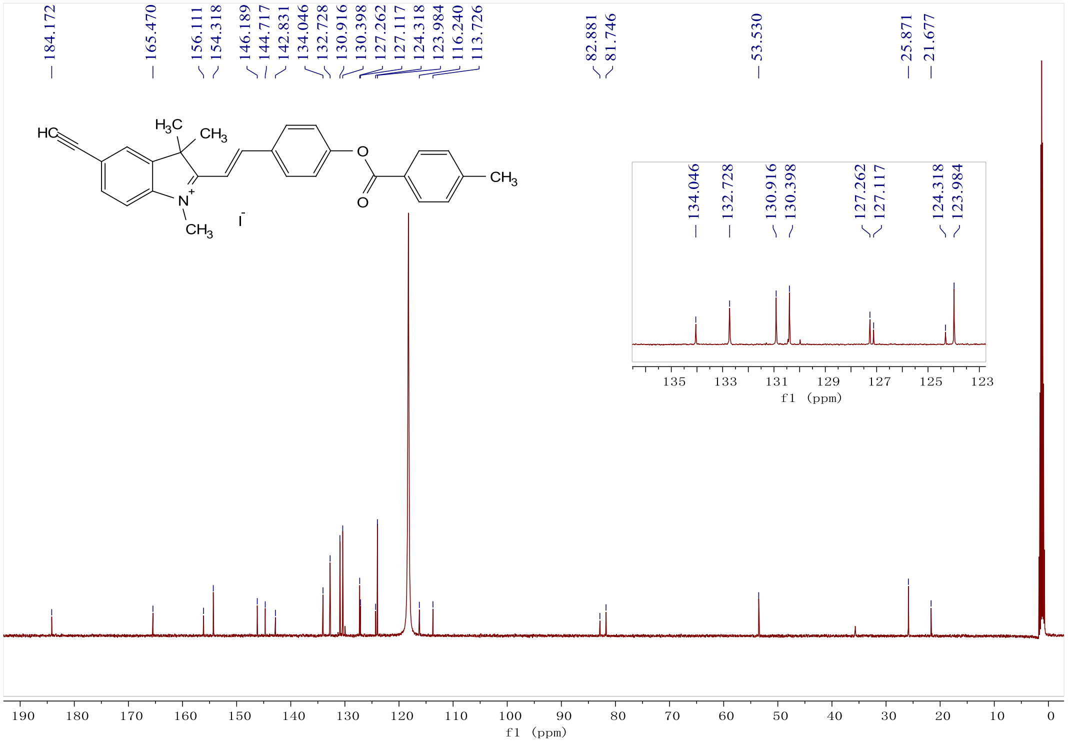

Figure S25. ^{13}C NMR spectrum (125 MHz) of ET-3 in CH_3CN-d_3. δ 184.17, 165.47, 156.11, 154.32, 146.19, 144.72, 142.83, 134.05, 132.73, 130.92, 130.40, 127.26, 127.12, 124.32, 123.98, 116.24, 113.73, 82.88, 81.75, 53.53, 25.87, 21.68 ppm.

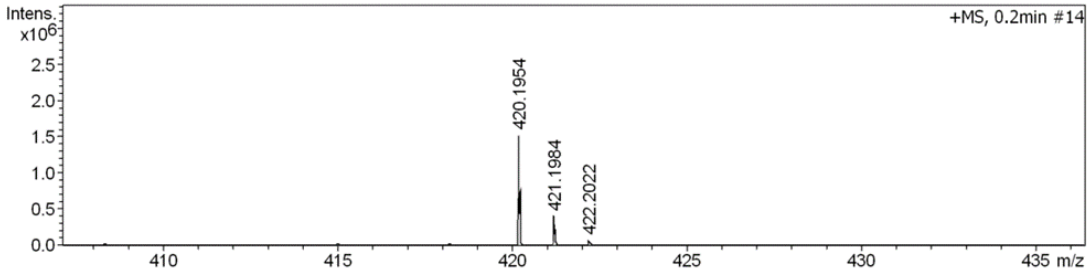

#	m/z	Res.	S/N	I	I %	FWHM
1	420.1954	23527	1771.2	1518416	100.0	0.0179
2	421.1984	21911	467.1	401088	26.4	0.0192
3	422.2022	16362	76.7	65988	4.3	0.0258

Meas. m/z	#	Ion Formula	m/z	err [ppm]	mSigma		Score	rdb	e⁻ Conf	N-Rule
420.1954	1	C29H26NO2	420.1958	0.9	28.9	1	100.00	17.5	even	ok

Figure S26. MS spectrum of ET-3.

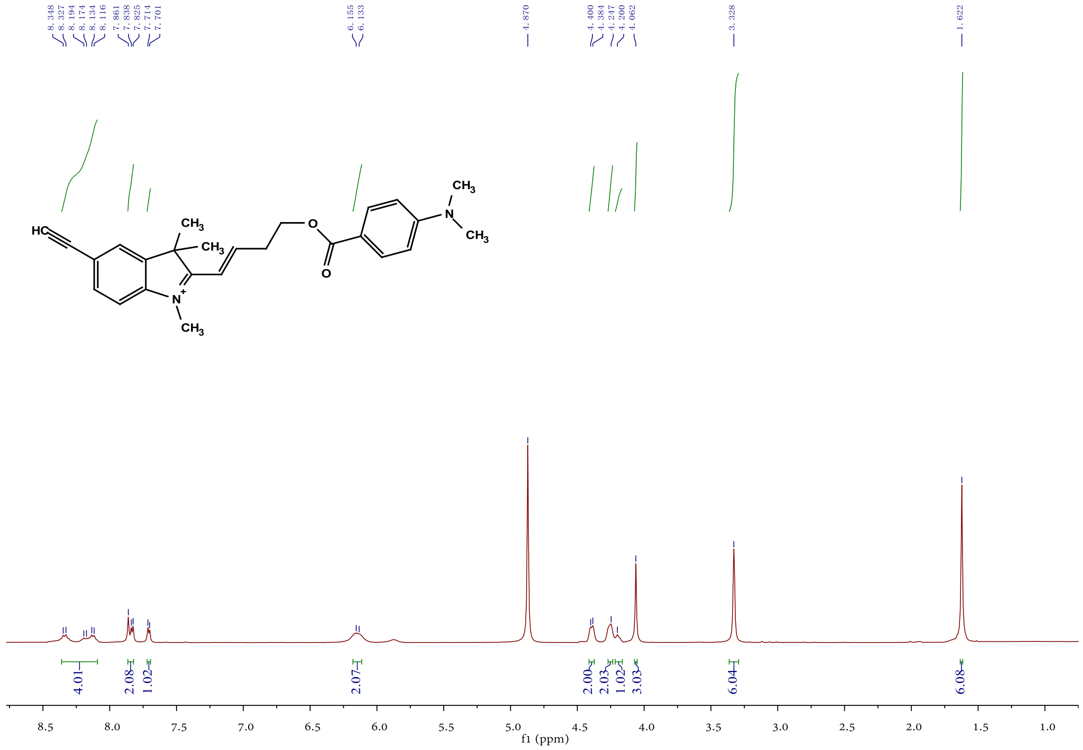

Figure S27. ^{1}H NMR spectrum (500 MHz) of ET-4 in CH_3OH-d_4.δ 8.415 – 8.069 (m, 4H), 7.868 – 7.818 (m, 2H), 7.708 (d, *J* = 5.3 Hz, 1H), 6.144 (d, *J* = 8.8 Hz, 2H), 4.392 (d, *J* = 6.7 Hz, 2H), 4.247 (s, 2H), 4.200 (s, 1H), 4.062 (s, 3H), 3.328 (s, 6H), 1.622 (s, 6H).

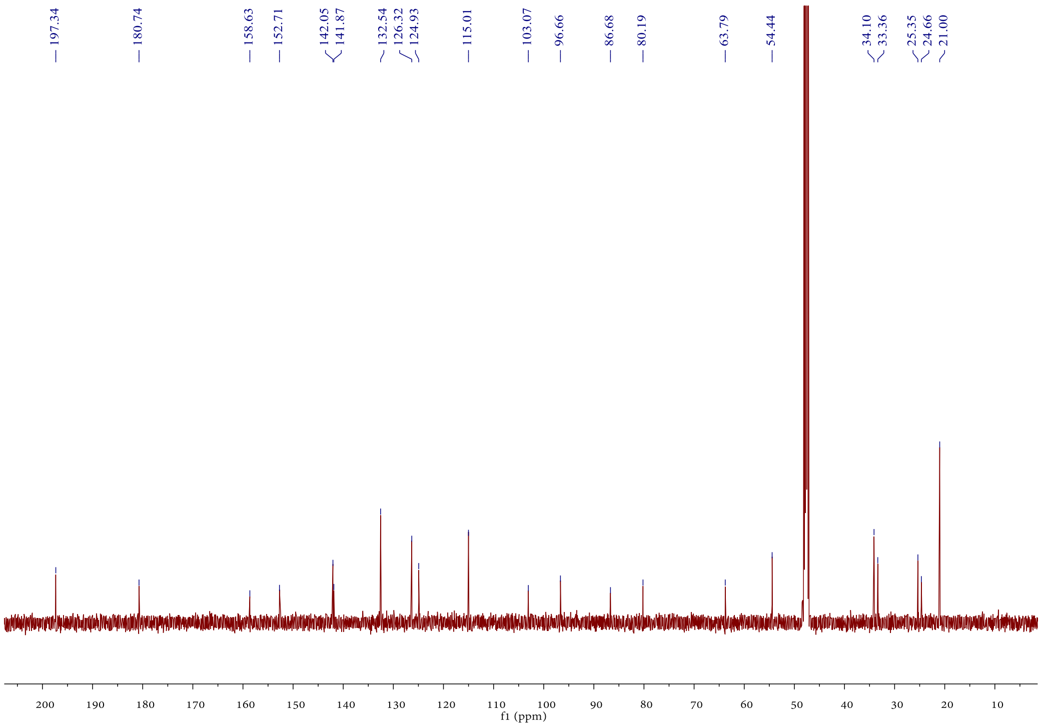

Figure S28. ^{13}C NMR spectrum (125 MHz) of ET-4 in CH_3OH-d_4. δ 197.34, 180.74, 158.63, 152.71, 142.05, 141.87, 132.54, 126.32, 124.93, 115.01, 103.07, 96.66, 86.68, 80.19, 63.79, 54.44, 34.10, 33.36, 25.35, 24.66, 21.00.

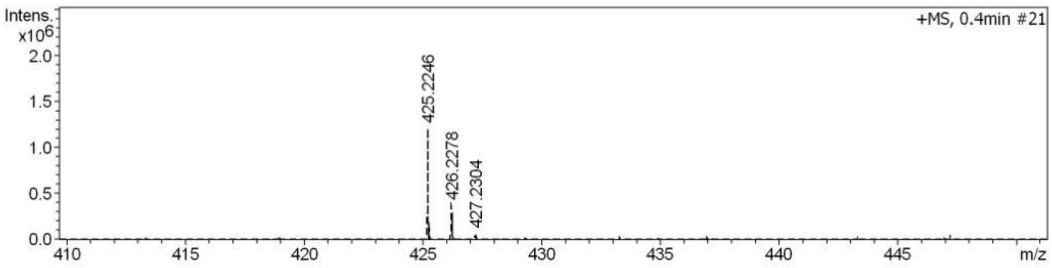

#	m/z	Res.	S/N	I	I %	FWHM
1	425.2246	21016	1166.3	1204808	100.0	0.0202
2	426.2278	16216	359.2	370436	30.7	0.0263
3	427.2304	13337	56.4	58104	4.8	0.0320

Meas. m/z	#	Ion Formula	m/z	err [ppm]	mSigma	#	Score	rdb	e⁻ Conf	N-Rule
425.2246	1	C26H30N2NaO2	425.2224	-5.4	3.9	1	100.00	15.5	even	ok

Figure S29. MS spectrum of ET-4.

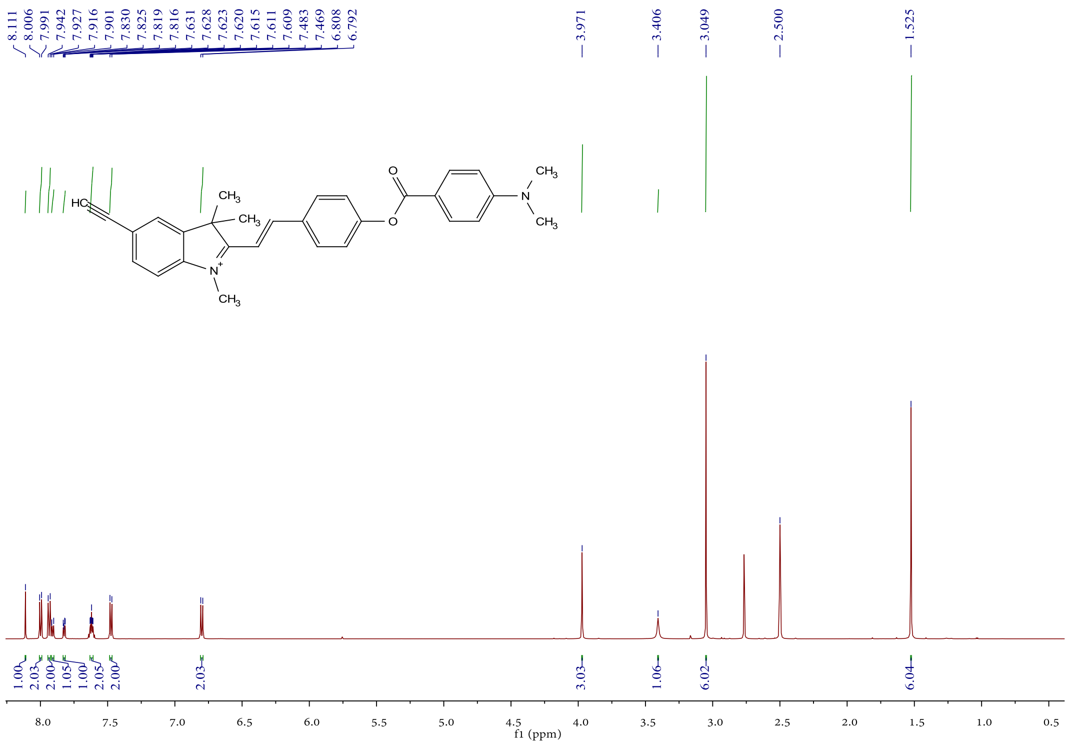

Figure S30. ^{1}H NMR spectrum (500 MHz) of ET-5 in DMSO-d_6. δ 8.111 (s, 1H), 7.999 (d, *J* = 5.7 Hz, 2H), 7.935 (d, *J* = 6.0 Hz, 2H), 7.908 (d, *J* = 5.8 Hz, 1H), 7.823 (dd, *J* = 4.0, 1.6 Hz, 1H), 7.620 (ddd, *J* = 4.1, 2.7, 1.0 Hz, 2H), 7.476 (d, *J* = 5.7 Hz, 2H), 6.800 (d, *J* = 6.1 Hz, 2H), 3.971 (s, 3H), 3.406 (s, 1H), 3.049 (s, 6H), 1.525 (s, 6H).

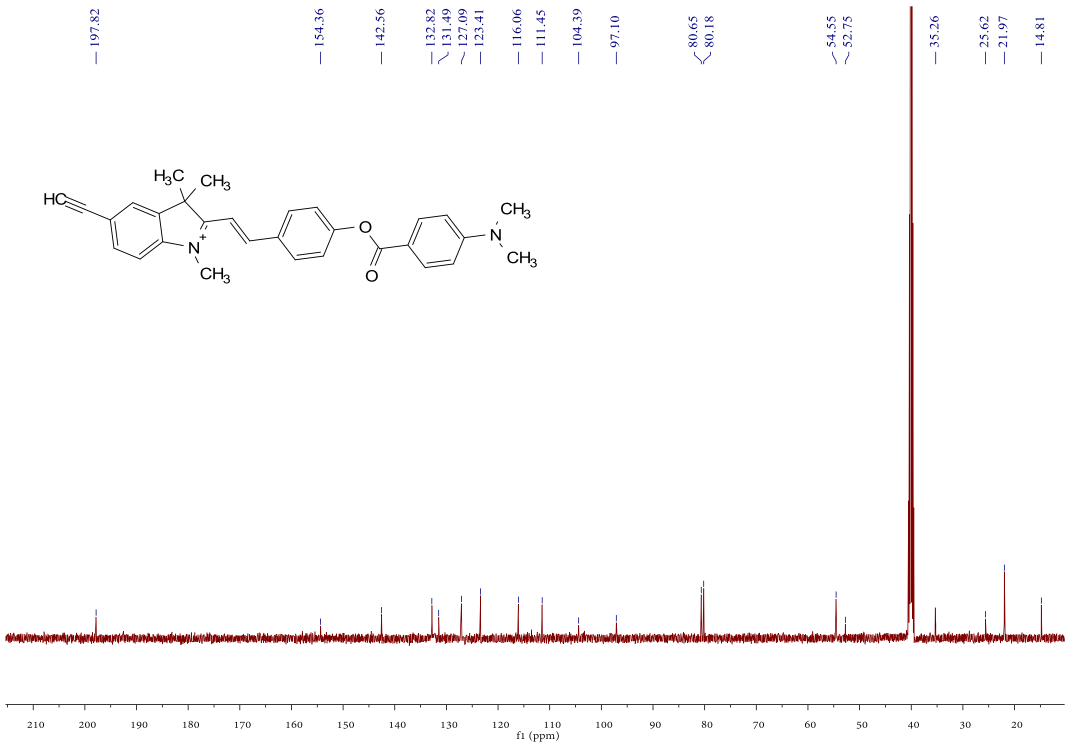

Figure S31. ^{13}C NMR spectrum (125 MHz) of ET-5 in DMSO. δ 197.82, 154.36, 142.56, 132.82, 131.49, 127.09, 123.41, 116.06, 111.45, 104.39, 97.10, 80.65, 80.18, 54.55, 52.75, 35.26, 25.62, 21.97, 14.81.

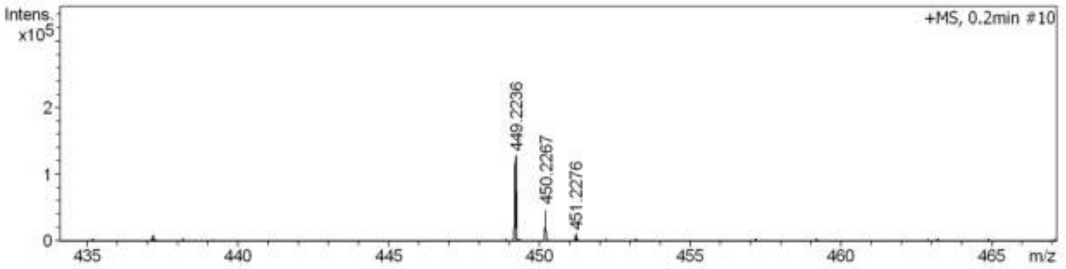

#	m/z	Res.	S/N	I	I %	FWHM
1	449.2236	16352	248.3	126532	100.0	0.0275
2	450.2267	17061	92.8	47272	37.4	0.0264
3	451.2276	15281	15.8	8052	6.4	0.0295

Meas. m/z	#	Ion Formula	m/z	err [ppm]	mSigma	#	Score	rdb	e⁻ Conf	N-Rule
449.2236	1	C30H29N2O2	449.2224	-2.9	19.3	1	100.00	17.5	even	ok

Figure S32. MS spectrum of ET-5.

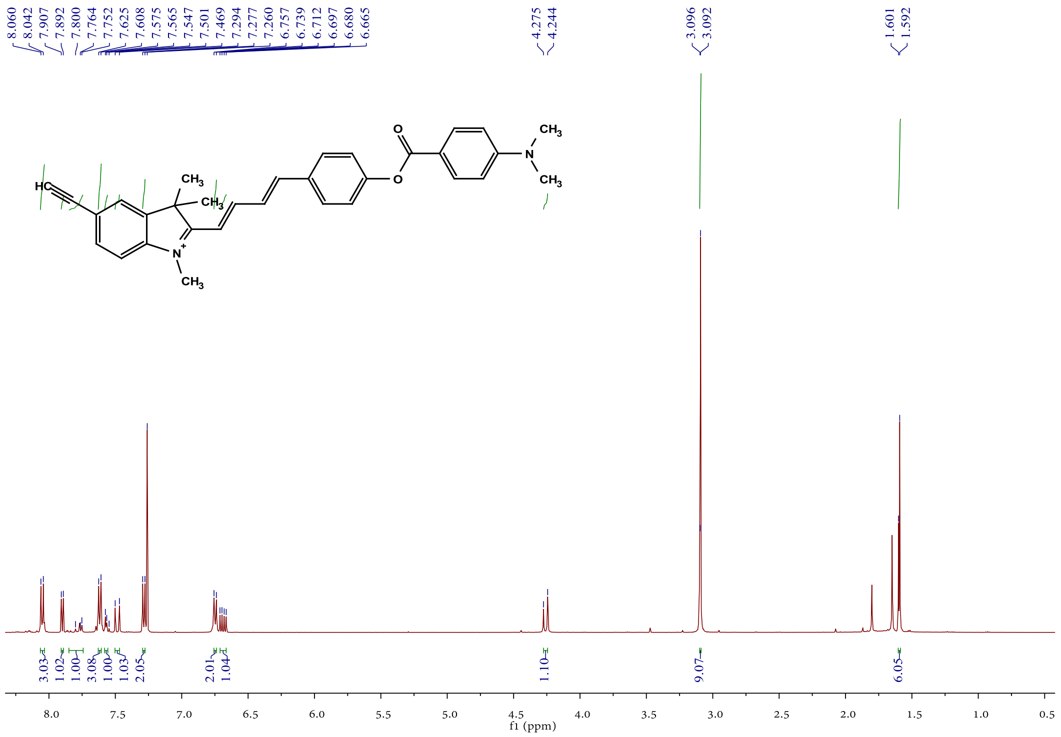

Figure S33. ^{1}H NMR spectrum (500 MHz) of ET-6 in $CHCl_3$-*d*$_1$.δ 8.076 (d, *J* = 7.2 Hz, 3H), 7.924 (d, *J* = 6.1 Hz, 1H), 7.801 (d, *J* = 19.1 Hz, 1H), 7.642 (d, *J* = 6.9 Hz, 3H), 7.595 (d, *J* = 4.2 Hz, 1H), 7.510 (d, *J* = 12.7 Hz, 1H), 7.310 (d, *J* = 6.8 Hz, 2H), 6.773 (d, *J* = 7.1 Hz, 2H), 6.713 (dd, *J* = 12.7, 6.1 Hz, 1H), 4.269 (s, 1H), 3.119 (d, *J* = 1.6 Hz, 9H), 1.621 (d, *J* = 3.6 Hz, 6H).

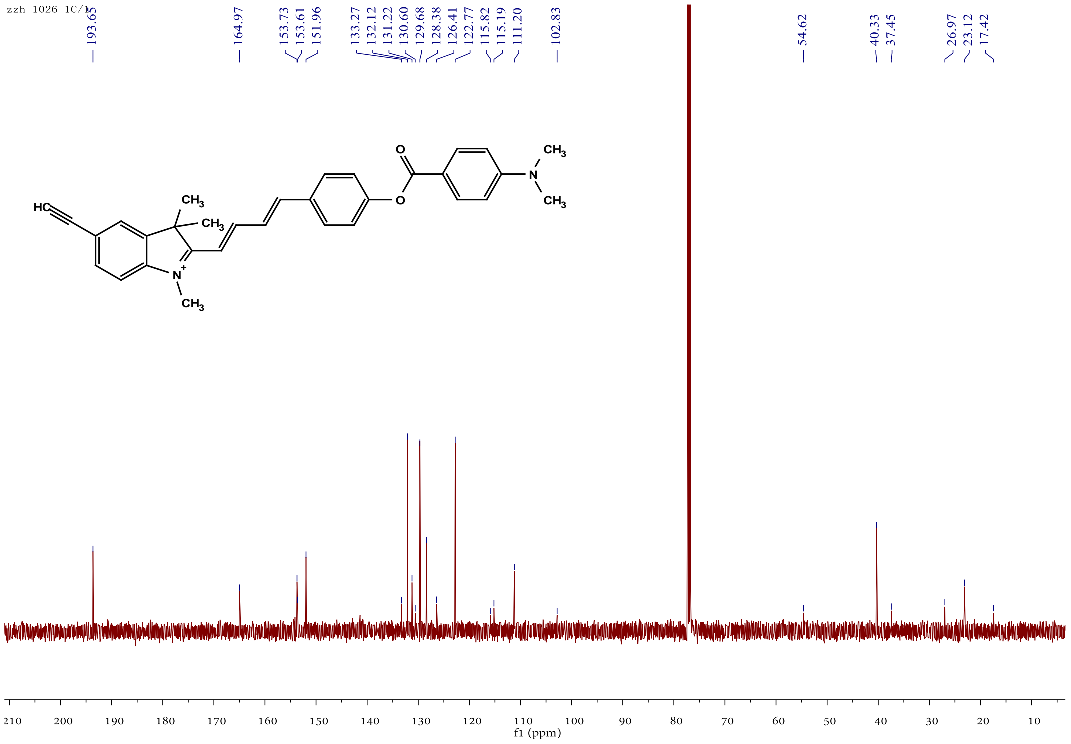

Figure S34. ^{13}C NMR spectrum (125 MHz) of ET-6 in $CHCl_3$-*d*$_1$. δ 193.65, 164.97, 153.73, 153.61, 151.96, 133.27, 132.12, 131.22, 130.60, 129.68, 128.38, 126.41, 122.77, 115.82, 115.19, 111.20, 102.83, 54.62, 40.33, 37.45, 26.97, 23.12, 17.42.

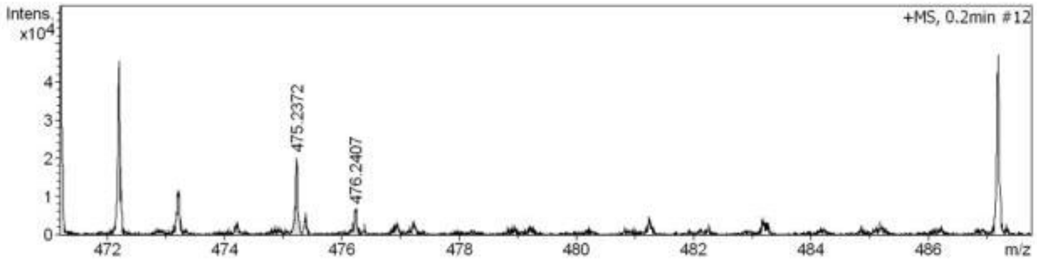

#	m/z	Res.	S/N	I	I %	FWHM
1	475.2372	14283	23.2	20120	100.0	0.0333
2	476.2407	13196	7.8	6724	33.4	0.0361

Meas. m/z	#	Ion Formula	m/z	err [ppm]	mSigma		Score	rdb	e^- Conf	N-Rule
475.2372	1	C28H27N8	475.2353	-4.0	27.4	1	60.72	19.5	even	ok
	2	C32H31N2O2	475.2380	1.7	35.4	2	100.00	18.5	even	ok
	1	C30H32N2NaO2	475.2356	-3.4	29.7	1	100.00	15.5	even	ok

Figure S35. MS spectrum of ET-6.

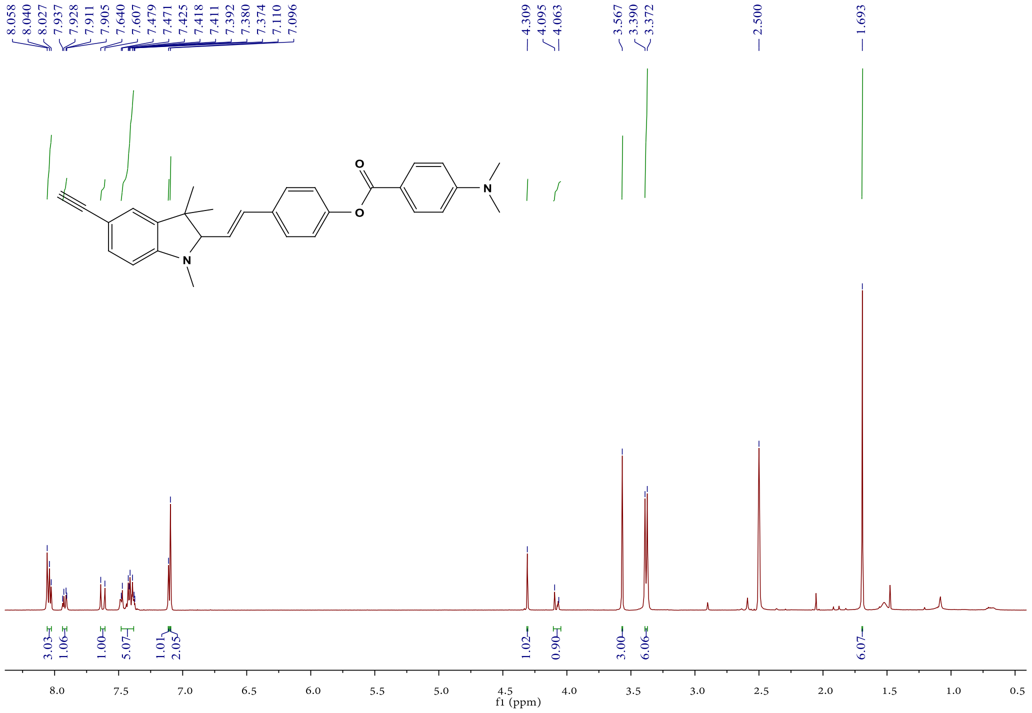

Figure S36. ^{1}H NMR spectrum (500 MHz) of ET-7 in DMSO-d_6.δ 8.058 – 8.025 (m, 3H), 7.938 – 7.905 (m, 1H), 7.624 (d, J = 13.0 Hz, 1H), 7.481 – 7.382 (m, 5H), 7.110 (s, 1H), 7.096 (s, 2H), 4.309 (s, 1H), 4.079 (d, J = 13.0 Hz, 1H), 3.567 (s, 3H), 3.381 (d, J = 7.1 Hz, 6H), 1.693 (s, 6H).

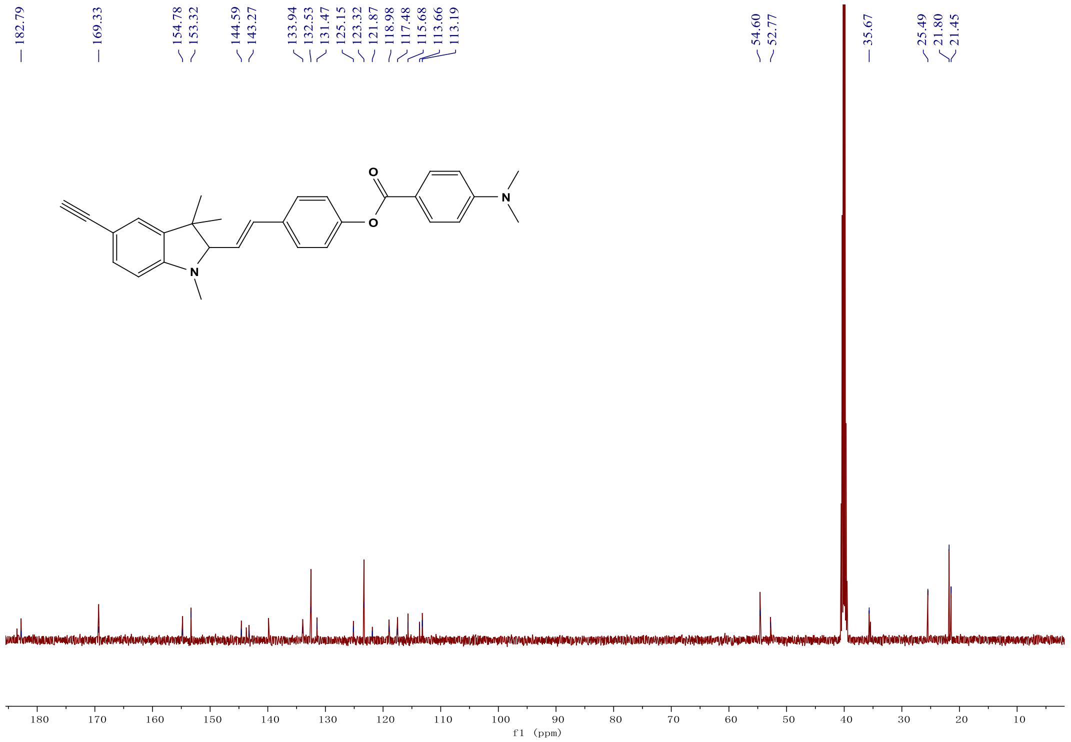

Figure S37. ^{13}C NMR spectrum (125 MHz) of ET-7 in DMSO-d_6. δ 182.79, 169.33, 154.78, 153.32, 144.59, 143.27, 133.94, 132.53, 131.47, 125.15, 123.32, 121.87, 118.98, 117.48, 115.68, 113.66, 113.19, 54.60, 52.77, 35.67, 25.49, 21.80, 21.45.

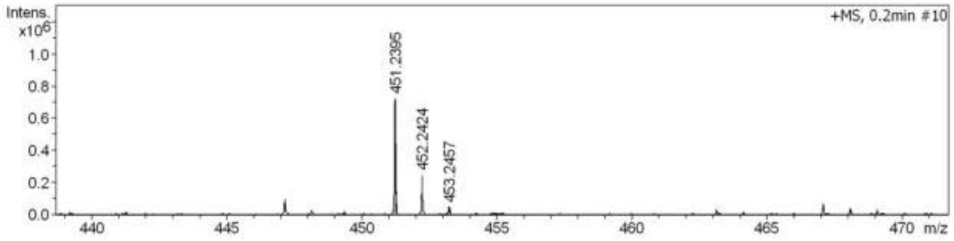

#	m/z	Res.	S/N	I	I %	FWHM
1	451.2395	19215	402.9	721548	100.0	0.0235
2	452.2424	14960	138.2	247312	34.3	0.0302
3	453.2457	10325	26.3	47016	6.5	0.0439

Meas. m/z	#	Ion Formula	m/z	err [ppm]	mSigma		Score	rdb	e⁻ Conf	N-Rule
451.2395	1	C30H31N2O2	451.2380	-3.3	5.5	1	100.00	16.5	even	ok

Figure S38. MS spectrum of ET-7.

3.3 The analytical performance of ET-R molecules.

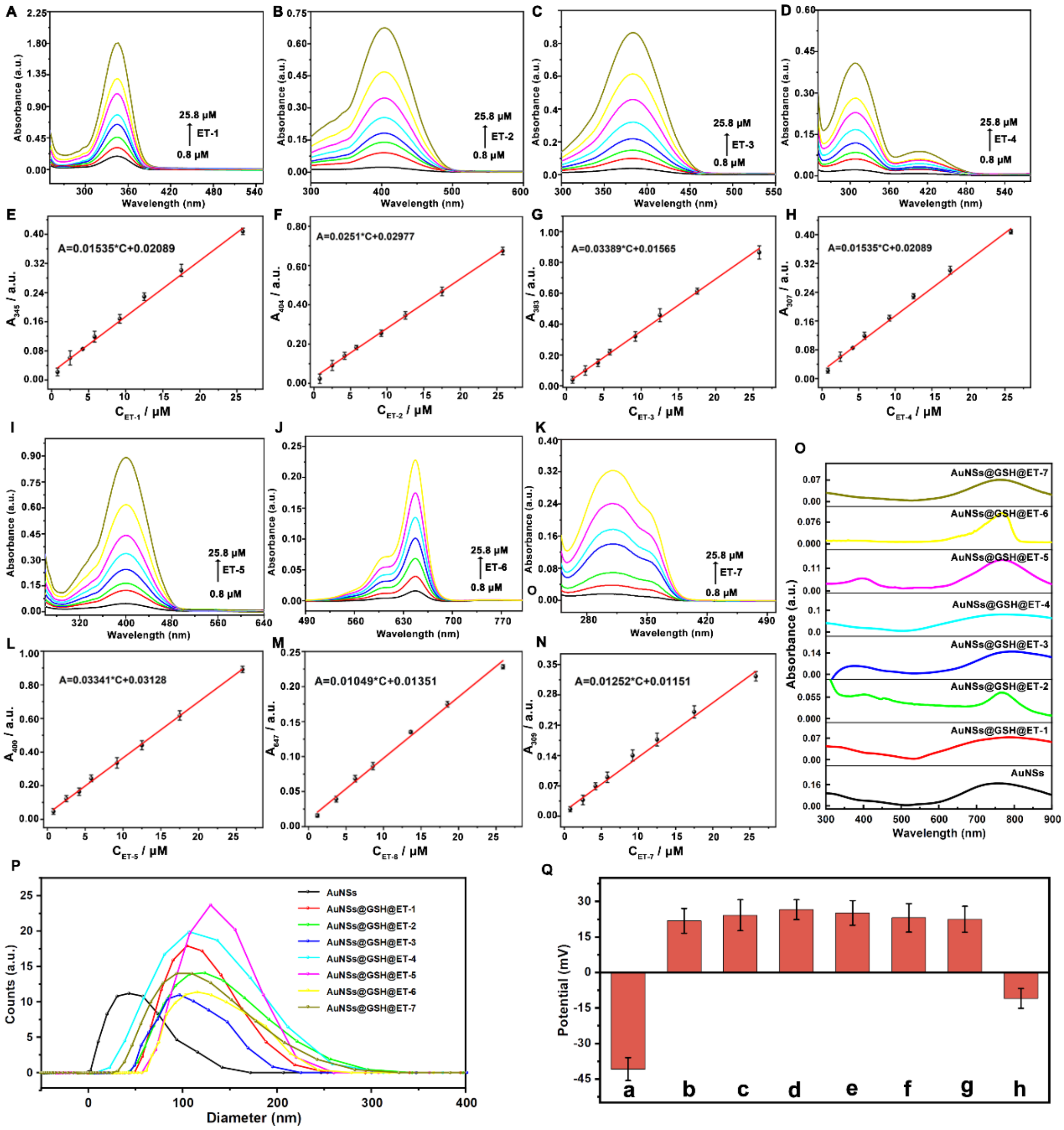

Figure S39. (A-D, I-K) Absorption spectra of (A) ET-1, (B) ET-2, (C) ET-3, (D) ET-4, (I) ET-5, (J) ET-6 and (K) ET-7 with different concentrations (0.8, 2.5, 4.2, 5.8, 9.2, 12.5, 17.5, 25.8 μM). (E-H, L-N) Linear calibration curves of of (E) ET-1, (F) ET-2, (G) ET-3, (H) ET-4, (L) ET-5, (M) ET-6 and (N) ET-7 with different concentrations (0.8, 2.5, 4.2, 5.8, 9.2, 12.5, 17.5, 25.8 μM). (n=5, S. E. M.) (O) Absorption spectra of AuNSs@GSH@ET-R (R=1-7), respectively. (P) Dynamic light scattering of AuNSs and AuNSs@GSH@ET-R probes (R=1-7). (Q) Zeta potential of (a) AuNSs and (b-h) AuNSs@GSH@ET-R probes (R=1-7). (n=5, S.E.M.)

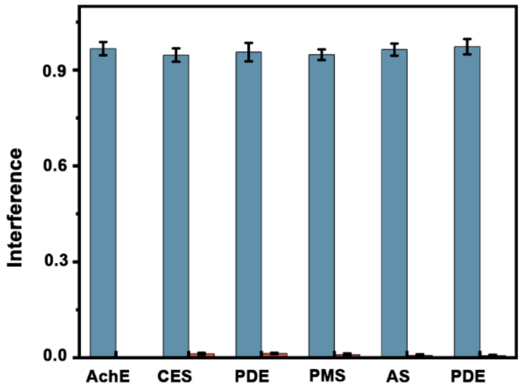

Figure S40. Selectivity (Blue bars) and competition (Red bars) tests for esterase (200 mU mL^{-1}) against AchE detection.

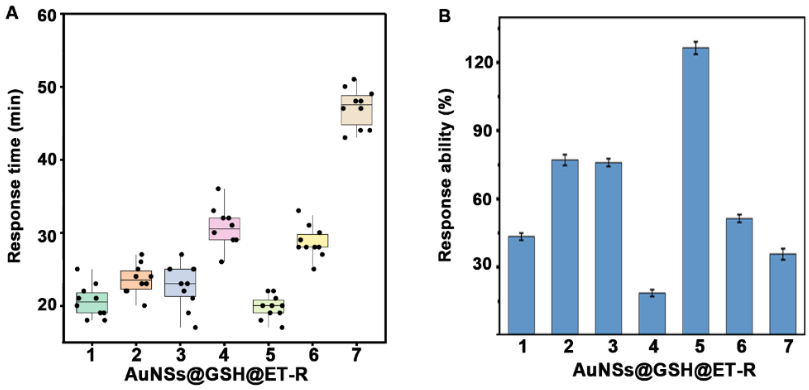

Figure S41. (A) The response time of AuNSs@GSH@ET-R (15.6±0.5 μg mL^{-1}) to AchE (500 mU mL^{-1}). (B) The response fluctuations of AuNSs@GSH@ET-R (R=1-7) (15.6±0.5 μg mL^{-1}) to AchE (500 mU mL^{-1}). The concentrations of ET-R loaded on AuNSs were 1.19±0.04 μM. (n=5, S. E. M.)

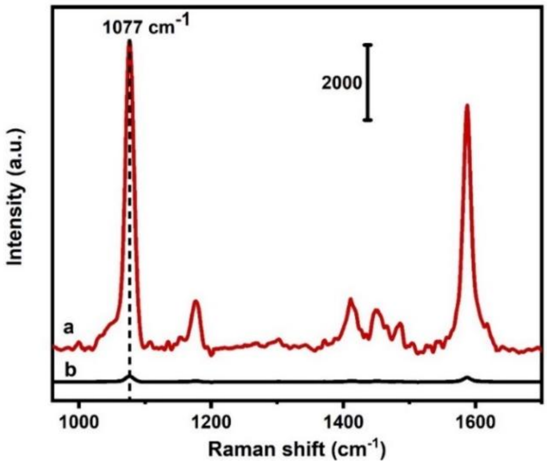

Figure S42. SERS spectrum of 4-Mercaptobenzoic acid (4-MBA) at the surface of AuNSs and Raman spectrum of an ethanol solution of 4-MBA (10 mM, 100 μL) dried onto a silicon wafer.

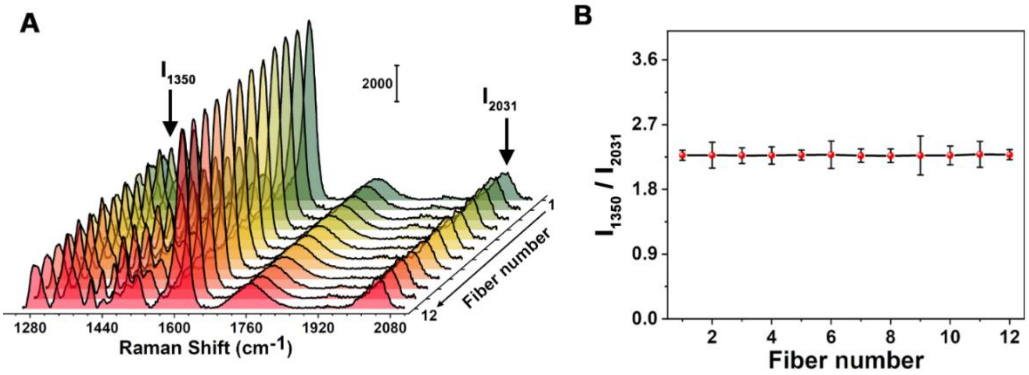

Figure S43. (A) SERS spectra obtained from 12 different TFs. (B) SERS intensity ratios I_{1350}/I_{2031} and acquired from 12 different TFs. Error bars equal to the standard deviations (n=25, S. E. M.).

3.4 The recognition mechanism of AuNSs@GSH@ET-R toward AchE.

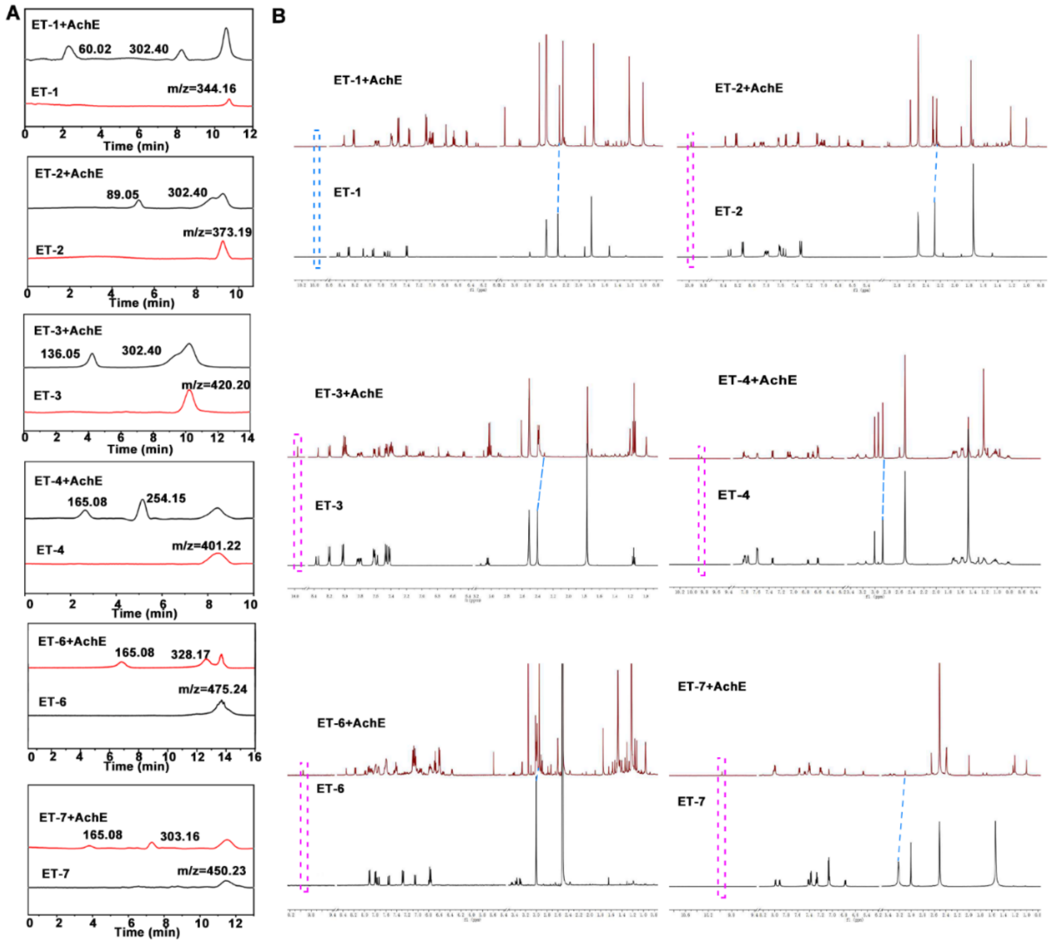

Figure S44. (A) LC-MS spectra (B) and partial ^{1}H NMR titration spectra of ET-R (5 mM) upon addition of AchE.

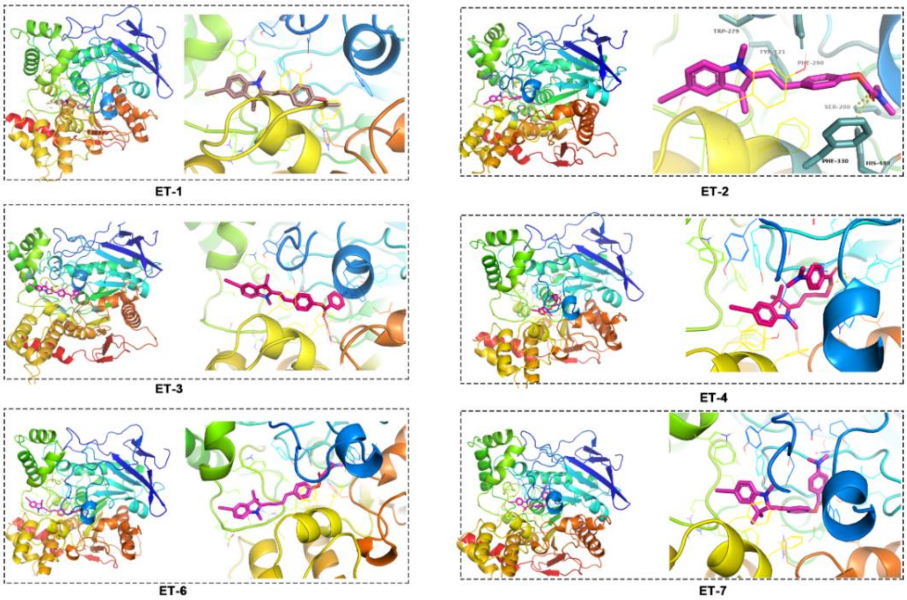

Figure S45. Molecular docking simulation of ET-R (R=1, 2, 3, 4, 6, 7) to AchE.

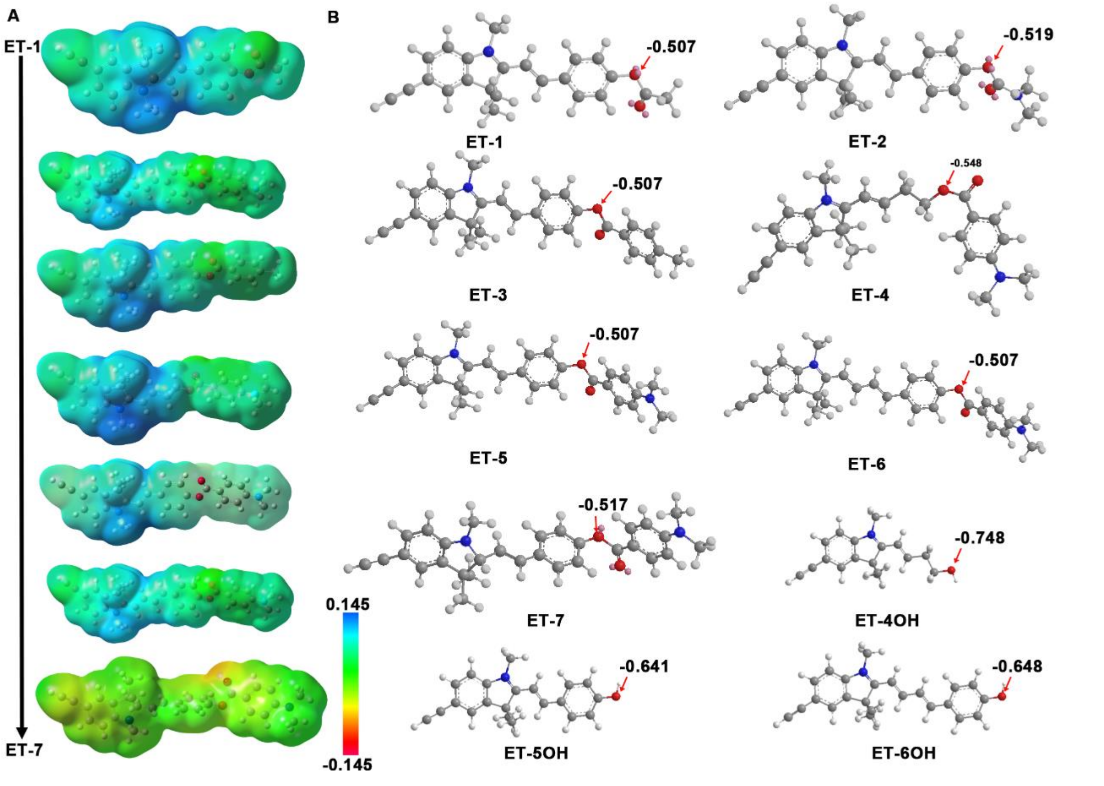

Figure S46. (A) The electron static potential (ESP) and (B) natural population analysis charges of ET-R (R=1-7).

3.5 Stability and biosafety of the develop AuNSs@GSH@ET-5.

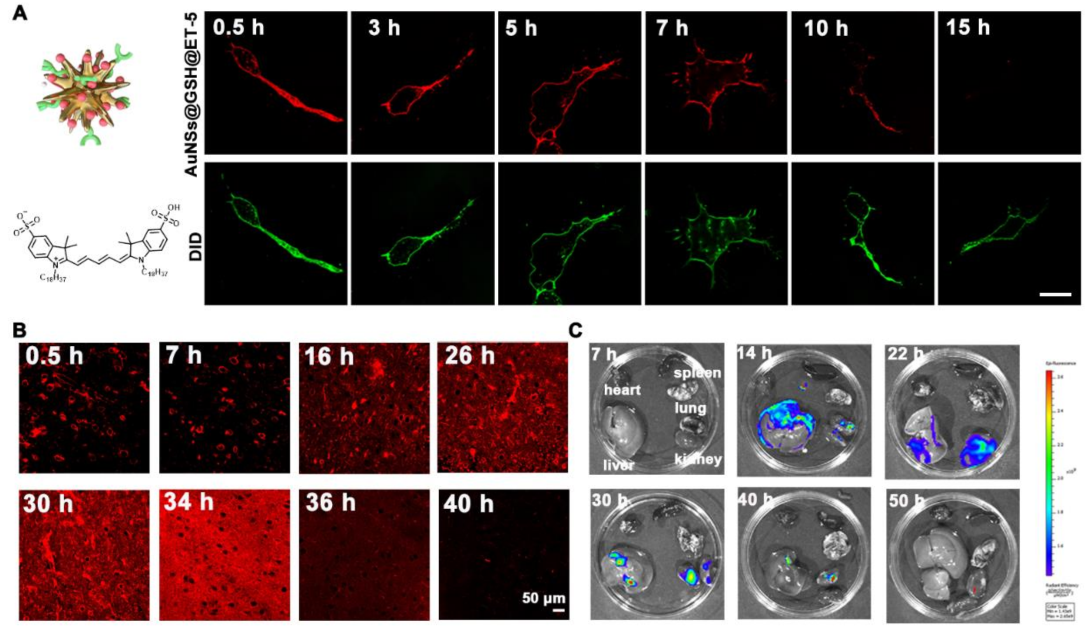

Figure S47. (A) Typical co-localization images of NSCs for different incubation times. (B) Confocal fluorescence images of brain slice after the developed probes were injected into the brain for different times. (C) Fluorescence imaging of organs obtained from the live mice after the developed probes were injected into the brain for different times.

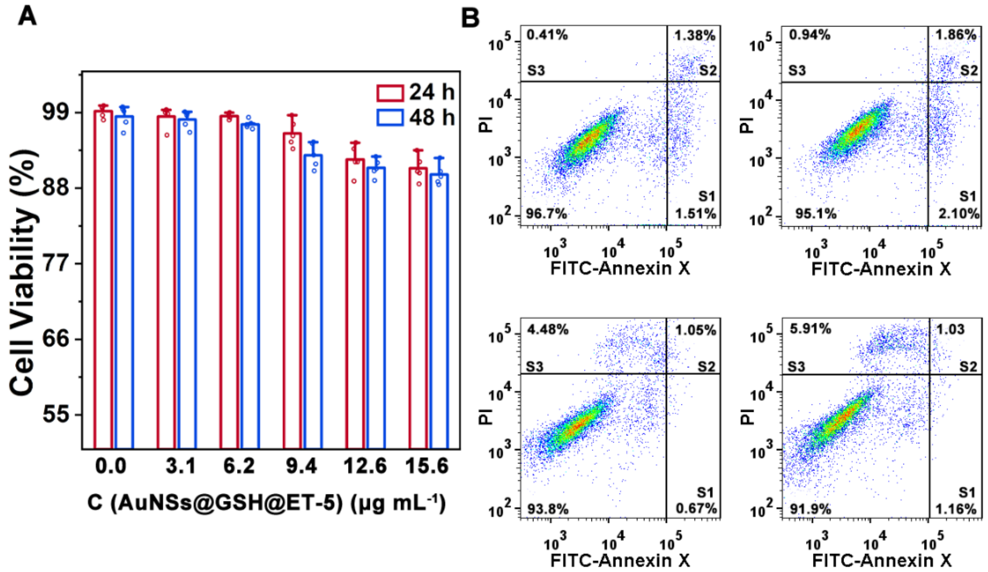

Figure S48. (A) Viabilities of NSCs after incubated with different concentrations of AuNSs@GSH@ET-5 for 24 h (red bars) or 48 h (blue bars), respectively. (B) NSCs incubated with different concentrations of AuNSs@GSH@ET-5 for 24 h, respectively. S1, S2, and S3 represent the regions of early apoptotic, late apoptotic and dead cells, respectively. (n=5, S. E. M,)

3.6 The ability of AchE to regulate the NSCs differentiation and proliferation.

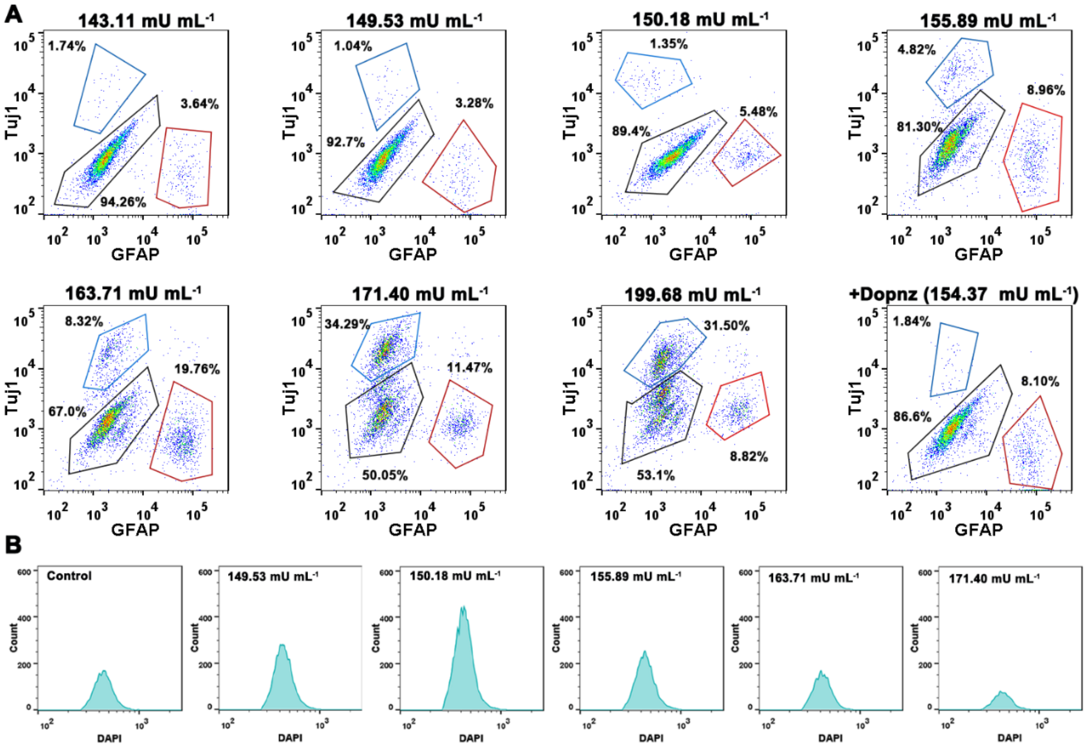

Figure S49. (A) The flow cytometry analysis of the NSCs differentiation to neuron (Tuj1, blue) and astrocyte (GFAP, red) with different activities of AchE. (B) Flow counting of NSCs accumulated within 2 min for NSCs with different activities of AchE.

3.7 SEM images of TFs with different angles.

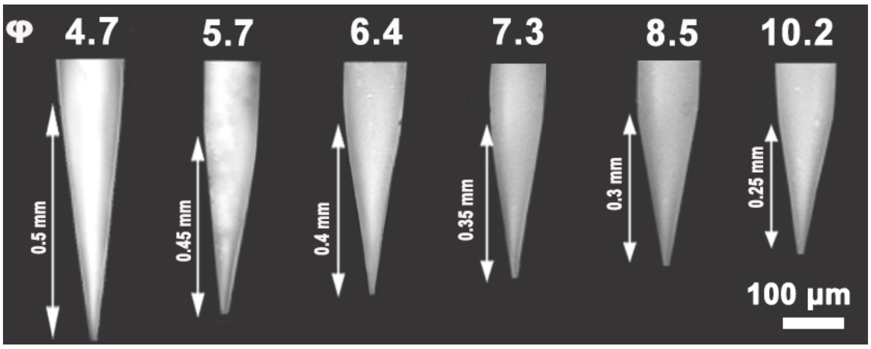

Figure S50. SEM images of TFs (NA 0.22) with different angles (φ 4.7°, 5.7°, 6.4°, 7.3°, 8.5° and 10.2°).

3.8 The co-localization and biocompatibility of the optical fiber array.

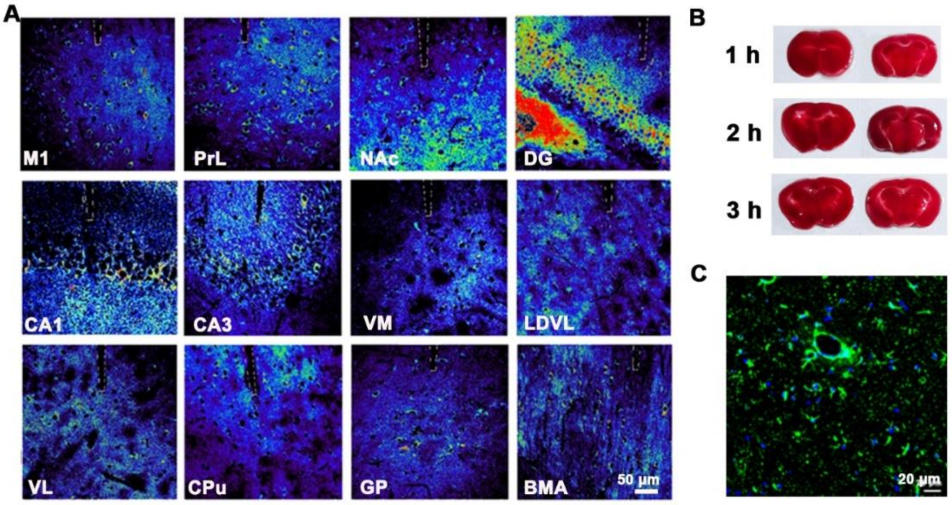

Figure S51. (A) Confocal images of brain tissues in different regions after implantation of multi-fiber microarray. (B) TTC staining of brain tissues cut from the live mouse brain after the fiber microarray was implanted into the live brain for different times. (C) Glial response in mouse brain tissue after implantation of a multi-fiber array.

3.9 The Raman spectra of AuNSs@GSH@ET-5 in 24 brain regions of AD mice

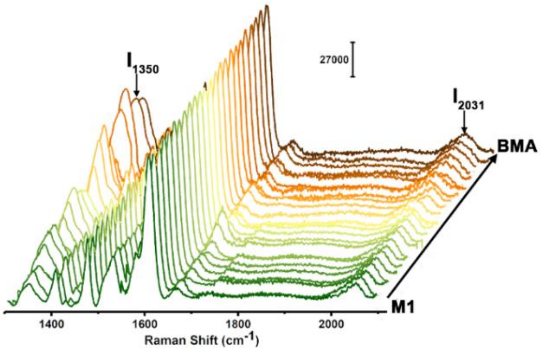

Figure S52. Raman spectra of AuNSs@GSH@ET-5 probe obtained by the developed Raman fiber photometry in 24 brain regions collected from 20 weeks AD mice.

4. Supplementary Tables

Table S1. Bands vibrational assignment for ET-5.

Raman shift / cm^{-1}	Vibration
1276	C-H bending vibration of benzen
1350	C-O stretching vibration
1474	C-H in-plane bending, C-C stretching
1504	C-N stretching, C-C stretching
1740	C=O stretching
2031	C≡C stretching

Table S2. List of brain regions targeted in the multi-fiber photometry experiments.

Medial anterior olfactory area	AOM
Posterior anterior olfactory area	AOP
Lateral orbital cortex	LO
Medial orbital cortex	MO
Prelimbic cortex	PrL
Infralimbic cortex	IL
Dorsal peduncular cortex	DP
Primary motor cortex	M1
Secondary motor cortex	M2
Primary somatosensory cortex	S1
Piriform cortex Pir; accumbens nucleus	NAc
Field CA1 of the hippocampus	CA1
Field CA2 of the hippocampus	CA2
Field CA3 of the hippocampus	CA3
Dentate gyrus	DG
Ventrolateral thalamic nucleus	VL
Ventromedial thalamic nucleus	VM
Ventral posterolateral thalamic nucleus	VPL
Laterodorsal thalamic nucleus, ventrolateral part	LDVL
Globus pallidus	GP
Caudate putamen	CPu
Basolateral amygdaloid nucleus, anterior part	BLA
Basomedial amygdaloid nucleus, anterior part	BMA

5. Supplementary references

1. Leeman, D.S., *et al.* Lysosome activation clears aggregates and enhances quiescent neural stem cell activation during aging. *Science* **359**, 1277-1282 (2018).
2. Vorhees, C.V. & Williams, M.T. Morris water maze: procedures for assessing spatial and related forms of learning and memory. *Nat. Protoc.* **1**, 848-858 (2006).
